# Risk of Hemolytic Uremic Syndrome Related to Treatment of *Escherichia coli* O157 Infection with Different Antimicrobial Classes

**DOI:** 10.3390/microorganisms9091997

**Published:** 2021-09-21

**Authors:** Rajal K. Mody, Robert M. Hoekstra, Magdalena Kendall Scott, John Dunn, Kirk Smith, Melissa Tobin-D’Angelo, Beletshachew Shiferaw, Katie Wymore, Paula Clogher, Amanda Palmer, Nicole Comstock, Kari Burzlaff, Sarah Lathrop, Sharon Hurd, Patricia M. Griffin

**Affiliations:** 1 Enteric Diseases Epidemiology Branch, Division of Foodborne, Waterborne, and Environmental Diseases, National Center for Emerging and Zoonotic Infectious Diseases, Centers for Disease Control and Prevention, Atlanta, GA 30329, USA; rmody@cdc.gov (R.K.M.); mgallopavo@icloud.com (R.M.H.); magdalena.scott@mt.gov (M.K.S.); 2 Tennessee Department of Health, Nashville, TN 37243, USA; John.Dunn@tn.gov; 3 Minnesota Department of Health, Saint Paul, MN 55164, USA; Kirk.Smith@state.mn.us; 4 Georgia Department of Public Health, Atlanta, GA 30303, USA; Melissa.Tobin-DAngelo@dph.ga.gov; 5 Oregon Public Health Division, Portland, OR 97232, USA; Beletshachew.SHIFERAW@dhsoha.state.or.us; 6 California Emerging Infections Program, Oakland, CA 94612, USA; kwymore@ceip.us; 7 Connecticut Emerging Infections Program, Yale School of Public Health, New Haven, CT 06510, USA; paula.clogher@yale.edu (P.C.); Shurdyale@gmail.com (S.H.); 8 Maryland Department of Health, Baltimore, MD 21201, USA; amanda.palmer@umaryland.edu; 9 Colorado Department of Public Health and Environment, Denver, CO 80246, USA; nicole.comstock@state.co.us; 10 New York State Department of Health Emerging Infections Program, Albany, NY 14202, USA; kari.burzlaff@health.ny.gov; 11 New Mexico Emerging Infections Program, School of Medicine, University of New Mexico, Albuquerque, NM 87106, USA; SLathrop@salud.unm.edu

**Keywords:** hemolytic uremic syndrome, *Escherichia coli* O157, diarrhea

## Abstract

Treatment of Shiga toxin-producing *Escherichia coli* O157 (O157) diarrhea with antimicrobials might alter the risk of hemolytic uremic syndrome (HUS). However, full characterization of which antimicrobials might affect risk is lacking, particularly among adults. To inform clinical management, we conducted a case-control study of residents of the FoodNet surveillance areas with O157 diarrhea during a 4-year period to assess antimicrobial class-specific associations with HUS among persons with O157 diarrhea. We collected data from medical records and patient interviews. We measured associations between treatment with agents in specific antimicrobial classes during the first week of diarrhea and development of HUS, adjusting for age and illness severity. We enrolled 1308 patients; 102 (7.8%) developed confirmed HUS. Antimicrobial treatment varied by age: <5 years (12.6%), 5–14 (11.5%), 15–39 (45.4%), ≥40 (53.4%). Persons treated with a β-lactam had higher odds of developing HUS (OR 2.80, CI 1.14–6.89). None of the few persons treated with a macrolide developed HUS, but the protective association was not statistically significant. Exposure to “any antimicrobial” was not associated with increased odds of HUS. Our findings confirm the risk of β-lactams among children with O157 diarrhea and extends it to adults. We observed a high frequency of inappropriate antimicrobial treatment among adults. Our data suggest that antimicrobial classes differ in the magnitude of risk for persons with O157 diarrhea.

## 1. Introduction

Shiga toxin-producing *Escherichia coli* O157 (O157) infection is the leading cause of postdiarrheal hemolytic uremic syndrome (HUS). Some [1,2,3], but not all, studies have found that patients treated with antimicrobials for O157 diarrhea were more likely to develop HUS, so many experts advise avoidance of antimicrobials [4]. Proposed mechanisms of harm include antimicrobial-triggered bacterial stress response (that induces bacteriophages encoding Shiga toxin genes (*stx*) to enter a lytic stage with production of phage-encoded proteins, cell lysis, and release of Shiga toxin and *stx*-encoding phage), direct release of preformed Shiga toxin from bacterial lysis, or alteration of the gastrointestinal flora [5]. However, antimicrobials differ by class in their ability to induce these mechanisms [6,7,8,9,10,11,12].

Definitive proof of this risk has been elusive. Randomized controlled trials have been considered too dangerous and difficult because events leading to HUS have often started by the time a diagnosis is made. This leaves data from observational studies, which are hampered by the fact that sicker patients are probably both more likely to receive antimicrobials and to develop HUS. Furthermore, variation in antimicrobial classes, dosing, and timing limits the ability of observational studies to accurately assess risk.

In 2016, Freedman et al., published a comprehensive meta-analysis that assessed associations between antimicrobial treatment and the development of HUS. In a sub-analysis restricted to five observational studies with lower risk of bias, they found that antimicrobial treatment of O157 diarrhea was associated with increased odds of HUS [13]. However, the meta-analysis did not account for how the severity of diarrheal illness could influence the decisions to use antimicrobials, and thereby confound associations with HUS, and it had limited ability to assess risks by specific antimicrobial class [14]. Although two methodologically robust observational studies (i.e., prospective or population-based, attempted to adjust for severity of illness, and used widely accepted criteria to define HUS) have been conducted among children [2,3], none have been reported that included adults, who also develop HUS.

Here we report on a large population-based observational study of O157 infections with adjustment for diarrheal illness severity conducted in 10 states to assess the risk of specific antimicrobial classes leading to development of HUS among persons of all ages.

## 2. Materials and Methods

### 2.1. Study Participants

The Foodborne Diseases Active Surveillance Network (FoodNet) conducts active population-based surveillance for O157 infections in seven states (Connecticut, Georgia, Maryland, Minnesota, New Mexico, Oregon, Tennessee) and selected counties in California, Colorado, and New York [15]. All residents with laboratory-confirmed O157 infection were eligible for inclusion. The start of the 4-year study varied by state, from January 2006 through January 2007. Laboratory confirmation was defined as isolation of O157 from a stool specimen and confirmation that it expressed the H7 antigen, produced Shiga toxin, or carried a *stx* gene.

An infection was excluded if the patient (or guardian) did not speak English or Spanish, did not report diarrhea, or could not be contacted by 10 telephone attempts within 45 days after collection of the specimen that yielded O157. All enrolled patients (or parent or legal guardians of patients < 18 years old) provided informed consent and patients 7–17 years old provided assent. The study was approved by Institutional Review Boards of the CDC and each FoodNet site.

### 2.2. Outcomes

We defined confirmed HUS as including all of these abnormalities during the first 10 days of illness: (1) hemoglobin or hematocrit below age- and gender-specific thresholds [16], (2) fragmented erythrocytes on peripheral blood smear, (3) platelets < 150 × 10^9^/L, and (4) serum creatinine ≥ 88.4 µmol/L if <13 years old or ≥132.6 µmol/L if ≥13 years old. For the primary analyses, data values not identified through medical record abstraction were assumed to be normal. Suspected HUS was defined as an illness diagnosed by a treating clinician as HUS or thrombotic thrombocytopenic purpura (TTP) in a patient with fragmented erythrocytes on peripheral blood smear, without other data available required for confirmed case classification. We included TTP in this definition because clinicians sometimes incorrectly use that term to describe thrombotic microangiopathy in the context of O157 infection, especially for adult patients. Day 1 of illness was defined as the day diarrhea began.

### 2.3. Data

In each state, trained staff administered a questionnaire by telephone < 45 days after stool specimen collection to each patient (or parent/guardian, hereafter not specified, but generally used for children) to obtain information on medical history, symptoms related to O157 infection, medications taken before and after diarrhea began, and related outpatient medical visits and hospitalizations. Staff used abstraction forms to collect information from all related outpatient medical encounters and hospitalizations, including data on signs and symptoms, clinician-diagnosed HUS or TTP, medications documented as administered or prescribed, and laboratory tests (forms available in Supplementary Materials).

Antimicrobial exposures during the first 3 or 7 days of illness were defined as documentation of oral, intramuscular, or intravenous antimicrobial administration or prescription in medical records. We conducted sensitivity analyses by assessing the impact of a broader definition of antimicrobial exposures: all exposures that were either documented in medical records or were patient-reported. Antimicrobial exposures after HUS diagnosis were disregarded. The antimicrobial classes individually evaluated were β-lactams, fluoroquinolones, nitroimidazoles (i.e., metronidazole), macrolides, and sulfonamide-containing agents. Trimethoprim-sulfamethoxazole and metronidazole accounted for all exposures to sulfonamide-containing agents and nitroimidazoles, respectively; we therefore refer specifically to these two agents.

### 2.4. Analyses

Analyses were performed using SAS 9.3 (code available on request). Differences between patient groups were assessed using Fisher’s exact test for categorical variables and Wilcoxon rank-sum test for continuous variables. Univariable logistic regression was used to further assess the association between each variable and confirmed HUS.

#### 2.4.1. Primary Models

To estimate the association between confirmed HUS and documented treatment with any antimicrobial or any agent in five antimicrobial classes during the first 3 or 7 days of illness, we developed multivariable logistic regression models to calculate adjusted odds ratios (aOR) with 95% confidence intervals (CI) and two-tailed *p* values. To provide a consistent frame of reference, each model was restricted to patients who either had documented exposure to the antimicrobial class of interest (possibly in conjunction with other antimicrobial agents) or had no documented exposure to any antimicrobial. Self-reported antimicrobial exposures that could not be verified by medical records were ignored (i.e., not considered exposures) in these primary models. To adjust for possible confounders, all variables (except initial white blood cell count (WBC) given the large amount of missing WBC data) associated with confirmed HUS by univariable logistic regression (*p* < 0.05) were included as covariates. For antimicrobial classes commonly prescribed in combination among patients in this study, we included exposure to the other antimicrobial class as a covariate in calculating the odds ratio for each antimicrobial class. Goodness of fit of each model was evaluated by the Hosmer–Lemeshow test. We assessed if associations between any antimicrobial and HUS varied by age quartile. These primary models excluded patients with suspected HUS because classifying these patients as either having or not having HUS could bias the observed associations. Imputation was not performed for missing covariates; thus, patients missing a value for any variable in the model were excluded.

#### 2.4.2. Secondary Models

To assess the robustness of the primary models, we performed a sensitivity analysis using secondary models that included the same covariates as the primary models, but different definitions of HUS and antimicrobial exposure. We also evaluated the impact of including the earliest recorded WBC count during days 1–10 of illness, limited to on or before the day of HUS diagnosis, as a covariate. These models evaluated three ways of handling suspected HUS cases (excluding them, treating them as not having HUS, and treating them as having HUS), two antimicrobial exposure definitions (documented, and documented plus patient-reported), and excluding or including WBC count. We also repeated all these analyses on the subset of patients with complete information for laboratory tests used to define confirmed HUS (i.e., values of these tests were not assumed to be normal when any of these data were not identified through medical record abstraction). No WBC count was available for 466 (36%) patients or was available only for dates after HUS was diagnosed; for models including WBC as a covariate, we assumed these patients had a WBC value < 17.2 × 10^9^/L (the 75th percentile among patients with confirmed HUS).

## 3. Results

### 3.1. Participants and Outcomes

**Figure 1:**
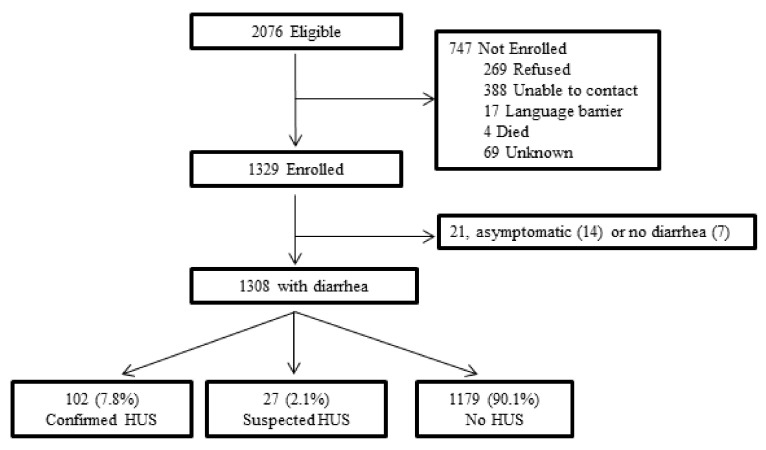
Diagram of study enrollment. An infection was excluded if the patient (or guardian) did not speak English or Spanish, did not report diarrhea, or could not be contacted by 10 telephone attempts within 45 days after collection of the specimen that yielded O157. The 747 persons who were not enrolled were similar to the 1329 enrolled patients with respect to age and sex: among those enrolled, median age was 15 years (IQR 5–40) and 52% were female; among those not enrolled, median age was 16 years (IQR 7–36) and 53% were female.

Of 2076 eligible persons, 1329 (64%) were enrolled and 1308 were eligible for analyses (Figure 1). Subjects’ ages ranged from 2 months to 91 years (median, 15 years; interquartile range, 5–40 years); 52% were female; 102 (7.8%) had confirmed HUS, and 27 (2.1%) had suspected HUS. Two of 578 hospitalized patients died: a child with confirmed HUS and an adult with neither confirmed nor suspected HUS. Patients with confirmed and suspected HUS were similar in demographic and clinical characteristics (Table 1).

### 3.2. Factors Associated with Disease Severity and Antimicrobial Exposures

Confirmed HUS was most common among children < 5 years old and decreased with increasing age. Other factors significantly associated with confirmed HUS were reported fever, vomiting, or use of acetaminophen anytime during the O157 illness, and seeking health care before day 4 of illness. Among 874 (67%) patients with WBC data, the frequency of confirmed HUS was greatest among patients with an initial WBC ≥ 13.5 × 10^9^/L during the first 10 days of illness (Table 2).

**Figure 2:**
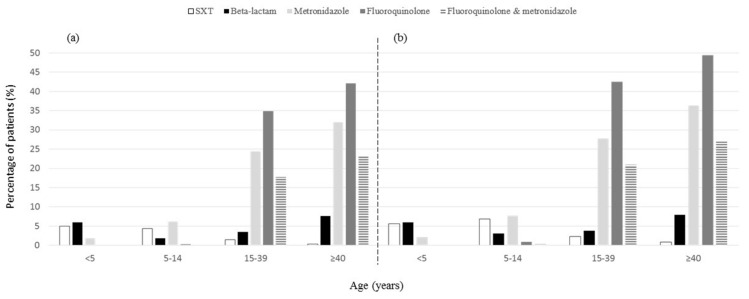
Percentage of patients treated with antimicrobials during the first 7 days of diarrhea caused by STEC O157, by age quartile and antimicrobial class. Panel (**a**) displays antimicrobial treatment documented in medical records. Panel (**b**) displays antimicrobial treatment documented in medical records or reported by the patient (or parent/guardian). Data on macrolides is not shown because only 20 patients received macrolides (documented among 12 (0.9%), reported among 8 (0.6%) more); documented macrolide exposure was greatest among children < 5 years old (1.3%) and lowest among adults ≥ 40 years old (0.3%). Abbreviation: SXT, trimethoprim-sulfamethoxazole.

Overall, 406 (31.0%) patients had documented antimicrobial exposure during the first 7 days of illness, with 225 (55%) of them exposed within the first 3 days. An additional 79 (6.0%) patients self-reported antimicrobial exposures during the first 7 days of illness that could not be verified in medical records. Several factors were associated with documented antimicrobial treatment (Table 3). The frequency of documented antimicrobial treatment varied greatly by age quartile: 12.6% (<5 years), 11.5% (5–14 years), 45.4% (15–39 years), 53.4% (≥40 years). The types of antimicrobials taken during the first 7 days of illness varied by age (Figure 2). Adults were most often treated with fluoroquinolones, metronidazole, or both. Children had greater exposure than adults to trimethoprim-sulfamethoxazole. The frequency of β-lactam use was similar across age quartiles (1.9–7.6%), with highest use among adults ≥ 40 years old.

### 3.3. Antimicrobial Treatment and HUS

#### 3.3.1. Unadjusted Analyses

Treatment with a β-lactam or trimethoprim-sulfamethoxazole was associated with a significantly elevated odds of developing confirmed HUS, and treatment with a fluoroquinolone was associated with decreased odds (Table 4). None of the 12 patients documented to have received a macrolide developed confirmed or suspected HUS; all 12 received azithromycin and two also received erythromycin. None of eight additional patients who self-reported taking a macrolide (azithromycin (7 patients), clarithromycin (1 patient)) developed confirmed or suspected HUS. Treatment with any antimicrobial during the first 7 days was not associated with confirmed HUS.

#### 3.3.2. Primary Adjusted Analyses

Of the 1281 patients included in the primary analyses (after removing 27 with suspect HUS), 1155 (90.2%) had complete information for all model covariates (age, time to healthcare presentation, and reported vomiting, fever, and acetaminophen use). The 1155 patients were slightly younger than those excluded due to missing data, but similar with respect to frequency of HUS and antimicrobial exposures (Supplementary Table S1). The total number of patients included in each adjusted model varied by the specific antimicrobial class assessed because of the varying number of patients excluded for taking only antibiotics other than those of the class of interest. After adjustment, the only association that remained was the elevated odds of confirmed HUS among patients treated with β-lactams within the first 7 days of illness (aOR 2.80, 95% CI 1.14–6.89) (Table 4). There was no significant effect modification by age quartile of any of the associations between antimicrobial classes and HUS. The directionalities of adjusted associations were similar for antimicrobial exposures during the first 3 days of illness; these did not reach statistical significance (Table 5).

#### 3.3.3. Secondary Models

In all secondary models, the odds of developing HUS was elevated among persons treated with β-lactams during the first 7 days of illness; these associations were statistically significant in all models except those most biased towards the null and least powered (Table 6). The analyses more biased towards the null were those that treated suspect HUS cases as not having HUS and only relied on documented antimicrobial exposures. The least-powered analyses were those that excluded patients missing data for any test used to define HUS. None of the other antimicrobial classes were significantly associated with HUS in any secondary model (Supplementary Tables S2–S4).

## 4. Discussion

We have reported on a very large, population-based, observational study designed to assess the association between treatment of O157 diarrhea with agents in specific antimicrobial classes and the development of HUS among patients of all ages. Our findings strongly suggest that the magnitude of risk varies by antimicrobial class. Two findings have implications for clinical practice. First, after adjusting for severity of illness and age, treatment with a β-lactam antimicrobial during the first 7 days of illness was associated with an increased odds of developing HUS. Others have described this for children with O157 diarrhea; our findings confirm this and provide the strongest evidence that this risk applies to persons of all ages. Second, despite longstanding clinical guidelines to avoid empiric antimicrobial treatment of most patients with suspected infectious diarrhea [17], the frequency of such treatment was high among adults with O157 diarrhea, a finding that can inform antimicrobial stewardship practices.

In the absence of randomized controlled trials, one means of demonstrating risk associated with treatment is through the emergence of a clear and consistent signal across a series of observational studies coupled with laboratory and animal experiments indicating biological plausibility of harm. Twenty years ago, experts considered previous observational studies suggesting that at least some antimicrobial classes may carry risks, combined with a lack of clearly demonstrated benefit of antimicrobial treatment, sufficient to advise clinicians not to treat O157 diarrhea with antimicrobial agents [17]. With the addition of our study, the data are compelling that treatment of O157 diarrhea with a β-lactam antimicrobial during the first 7 days of illness increases the risk of HUS. Two smaller studies that adjusted for disease severity found increased risk of HUS among children treated with β-lactams during the first 7 days of O157 diarrheal illness [2,18]. However, the final report from one of these studies did not provide severity-adjusted risk estimates by antimicrobial class [3]. A study among patients of all ages found that treatment with β-lactams, but not other antimicrobials, was associated with HUS; however, although the analysis was adjusted for age and severity of illness, clinical criteria were not used to define HUS, antimicrobial exposures were not verified, and timing of antimicrobial exposures in relation to onset of diarrhea was not ascertained [19].

The highest risk period of β-lactam exposure following onset of O157 diarrhea remains uncertain. Although we did not find evidence of increased odds of HUS associated with antimicrobial treatment during the first 3 days of illness, only about half of observed antimicrobial exposures in our cohort occurred during the first three days, limiting statistical power. Another study observed a greater proportion of antibiotic exposures during the first 3 days; it found the magnitude of risk from β-lactam exposure to be greater in the first 3 days than the first 7 days [2].

The elevated risk of HUS following β-lactam treatment is supported by the sensitivity analyses afforded by our secondary models. Use of the model with our most inclusive HUS case definition (assumed missing laboratory results used to define HUS were normal and treated suspect and confirmed HUS as equivalent) is supported by the clinical similarity between suspect and confirmed cases. This model likely provides the most accurate estimates. Thus, our models suggest the best estimate for the association between β-lactam treatment and HUS is an odds ratio of 2.80 to 3.90. The secondary models that did not yield significant associations with β-lactam treatment were the least statistically powered and were intentionally biased towards the null.

β-lactam antimicrobials, especially at sub-inhibitory concentrations, can increase Shiga toxin production and release by O157 bacteria in vitro [6,7,9,20]. The specific mechanism(s) for these effects have not been clearly defined. Although β-lactam agents are not strong inducers of the SOS stress response, their harmful effects may also involve induction of toxin production through other mechanisms or direct release of preformed Shiga toxin and bacteriophage from increased bacterial cell wall permeability [6,8]. Because Shiga toxin 1 may attenuate the more toxic effects of Shiga toxin 2 [21], it is noteworthy that sub-inhibitory concentrations of β-lactams can differentially increase production of Shiga toxin 2 and decrease production of Shiga toxin 1 in vitro [6]. Although the Shiga toxin profile (production of Shiga toxin 1, Shiga toxin 2, or both) may influence the effect of β-lactams on HUS risk, we chose to not include Shiga toxin profiles in our analyses because this information is typically not known when a decision is being made about whether to treat a patient’s diarrheal illness with antibiotics.

Our findings do not exonerate other antimicrobial agents. Sulfonamides, mainly trimethoprim-sulfamethoxazole, have long been suspected of increasing the risk of HUS [18,22]. We found the magnitude of risk associated with trimethoprim-sulfamethoxazole to be similar to that with β-lactams; the lack of statistical significance may reflect limited use of trimethoprim-sulfamethoxazole. In vitro data indicate that both trimethoprim-sulfamethoxazole and fluoroquinolones are strong inducers of Shiga toxin production via the SOS stress response [7,8,10,11,12,20] and animals with O157 infection experience worse outcomes when treated with fluoroquinolones [12,23]. A meta-analysis found an increased, but not statistically significant, odds of HUS among persons treated with trimethoprim-sulfamethoxazole or fluoroquinolones [13]. Because fluoroquinolones are rarely prescribed for young children, the age group at highest risk of HUS, residual confounding by age may explain the lack of significantly increased odds of HUS with these agents in our analysis. Investigators of Shiga toxin-producing *E. coli* (STEC) O104:H4 infections among children and adults reported a significantly reduced odds of HUS with fluoroquinolone treatment, but their failure to adjust for age makes these results difficult to interpret [24]. Others, like us, found that receipt of nitroimidazoles (e.g., metronidazole), was associated with an increased frequency of HUS that was not statistically significant [2]. In the absence of known benefit, the possibility of harm justifies avoidance of trimethoprim-sulfamethoxazole (and likely other sulfonamide-containing agents), fluoroquinolones, and nitroimidazoles among patients with O157 diarrhea.

Far less evidence supports the claim that other classes of antimicrobials can harm patients with O157 diarrhea. Considerable non-human experimental data suggest some antimicrobials might reduce the risk of HUS. Several in vitro studies have found that exposure to macrolides and to fosfomycin at a variety of concentrations reduces Shiga toxin production by O157 [8,11,25,26], and animal studies of these agents have reported improved, or not worse, O157 infection outcomes [12,23,26]. Although macrolides were not associated with a significantly reduced odds of HUS in our analysis, none of our small number of patients treated with a macrolide developed confirmed or suspected HUS. This differs slightly from other studies of O157 diarrhea. In one study, one of four children treated with azithromycin developed HUS [3]. In another, three (5%) of 63 children with HUS had been treated with azithromycin, compared with five (4%) of 125 children without HUS; the authors found no increased risk of HUS from treatment with bacteriostatic agents, including macrolides and other protein synthesis inhibitors [2]. Identifying antimicrobials that do not increase the risk of HUS is important because antimicrobial treatment is sometimes needed for concurrent infection or invasive STEC infections [27]. Others have proposed the macrolide azithromycin as a suitable agent for treatment of invasive STEC O80:H2 infection [27], and to reduce shedding of STEC O104:H4 [28]. Other agents that inhibit bacterial protein synthesis (e.g., rifaximin, tigecycline) and fosfomycin have also been mentioned as candidates [5].

Our data indicate that physicians often treat O157 diarrhea among persons ≥ 15 years old with antimicrobials, especially fluoroquinolones and metronidazole, frequently in combination. This suggests that previous observational studies that found associations between antimicrobial treatment and HUS among children did not have meaningful impact on the clinical management of diarrhea among adults. Empirical treatment can have important negative consequences beyond an increased risk of HUS, including triggering *Clostridioides difficile* infection and driving development of antimicrobial-resistant bacteria. We are unaware of any guideline that recommends empirically treating infectious diarrhea with fluoroquinolones, metronidazole, or both. The frequent use of these antimicrobials among patients in our cohort highlights an important opportunity for improved antimicrobial stewardship. The use of fluoroquinolones and metronidazole suggests that clinicians considered diagnoses other than O157 diarrhea highly likely, or were unaware that evidence does not support empirical treatment of most patients with presumed infectious diarrhea [4]. *E. coli* O157 diarrhea has been misdiagnosed as ischemic colitis, diverticular disease, peritonitis, and inflammatory bowel disease [29,30,31,32]; these conditions are sometimes treated with this combination of antimicrobials [33,34,35]. Misdiagnosis of O157 infections as these other conditions can contribute to poor outcomes and unnecessary procedures [30,31,32].

Although we recruited many O157 cases representative of the population, the retrospective nature of data collection introduced limitations, most notably incomplete ascertainment of laboratory variables needed to confirm HUS and to adjust for disease severity. The absence of WBC data for one-third of patients was an important limitation because a higher count early in the illness indicates a greater risk of HUS [36]. We attempted to account for this in secondary sensitivity analyses. Although our approach in sensitivity analyses of assigning all those having a missing WBC as having a value of <17.2 × 10^9^/L is a conservative assumption, it may have tended to artificially narrow the confidence intervals of analyses containing WBC as a covariate. Furthermore, considerable data was missing for several covariates in our adjusted models (fever, vomiting, acetaminophen use); this likely introduced some bias because patients excluded from adjusted analyses for missing data tended to be slightly older than those included.

In summary, we found that treatment of O157 diarrhea with β-lactams increases the odds of HUS. Because the mechanism of action is likely to be through production and release of toxin, these results indicate that this antimicrobial class should be avoided for highly virulent STEC infections (i.e., strains that produce Shiga toxin 2) [37]. Other antimicrobials might increase or decrease the risk of HUS. The increasing availability of culture-independent diagnostic tests might allow earlier diagnosis, which could decrease the frequency of the unnecessary antimicrobial treatment that was common clinical practice for diarrhea affecting adults. Improved diagnostics may also facilitate randomized controlled trials to study the effect of therapeutic agents administered before the events that lead to HUS are fully underway.

## Figures and Tables

**Table 1:** Demographic and clinical characteristics of confirmed and suspected HUS case-patients.

Characteristic	Suspected HUS ^a^ (*n* = 27)	Confirmed HUS ^a^ (*n* = 102)
Age, median (range) in years	4.3 (1.3–77.6)	4.3 (1.1–73.7)
Female, %	52	53.9
Median days to first medical encounter, (IQR)	2 (1–3)	2 (1–2)
Median days to HUS diagnosis, (IQR)	6.5 (4–9)	6 (5–7)
Highest WBC, median (IQR) in cells × 10^9^/L ^b^	18.3 (13.3–25.1)	15.9 (11.8–24.2)
Documentation of laboratory criteria used to define confirmed HUS ^c^:		
Elevated creatinine, %	38	100
Low hemoglobin or hematocrit, %	64	100
Platelet count < 150 × 10^9^/L, %	65	100
Red blood cell fragmentation, %	100	100

Abbreviations: HUS, hemolytic uremic syndrome; WBC, white blood cell count. ^a^ Confirmed HUS was defined as including all of the following abnormalities during the first 10 days of illness: (1) hemoglobin or hematocrit below age- and gender-specific thresholds,^21^ (2) fragmented erythrocytes on peripheral blood smear, (3) platelets <150 × 10^9^/L, and (4) serum creatinine ≥ 88.4 µmol/L if <13 years old or ≥ 132.6 µmol/L if ≥13 years old; values were assumed to be normal when any of these data were not identified through medical record abstraction. Suspected HUS was defined as an illness diagnosed by a treating clinician as HUS or thrombotic thrombocytopenic purpura in a patient with fragmented erythrocytes on peripheral blood smear, without other data available required for confirmed case classification. ^b^ During the first 10 days of illness; WBC data missing for 3 (11%) patients with suspected HUS and 13 (13%) with confirmed HUS. ^c^ One patient with suspected HUS had no creatinine value documented, another had no platelet count documented, and two had no hemoglobin or hematocrit documented; these patients were excluded when calculating the corresponding percentages. Of the 27 patients with suspected HUS, 18 (67%) had three of the four HUS-defining criteria, 7 (26%) had two, and 2 (7%) had only one.

**Table 2:** Frequency of confirmed HUS by demographic and clinical characteristics.

Characteristic	Frequency of Confirmed HUS, %	Confirmed HUS/Total Patients ^a,^ No./No.	*p* Value ^b^
All patients	7.8	102/1308	
Age in years			
<5	23.9	76/318	<0.001
5 to <15	10.5	34/323	
15 to <40	3.5	12/339	
≥40	2.1	7/328	
Sex			
Female	8.0	55/685	0.76
Male	7.6	47/622	
Race			
White	7.9	88/1113	0.98
Black	9	4/47	
Asian	4	1/27	
Other	8	3/40	
Unknown	7	6/81	
Fever^c^			
No	4.5	33/731	<0.001
Yes	12.2	63/516	
Vomiting ^c^			
No	2.3	16/686	<0.001
Yes	14.1	86/612	
Bloody stool ^c^			
No	6.8	10/148	0.74
Yes	7.9	91/1150	
Day of illness at time of first medical encounter			
1–3	9.2	77/837	0.03
4–7	5.8	23/396	
>7	3	2/75	
Acetaminophen use ^c^			
No	5.8	55/946	<0.001
Yes	13.5	40/296	
Nonsteroidal anti-inflammatory drug use ^c^			
No	7.2	77/1063	0.22
Yes	10.1	18/179	
Antimotility drug use ^c^			
No	8.2	78/955	0.15
Yes	5.4	15/278	
Initial WBC (×10^9^/L) ^d^			
3.9–10.3	4.9	15/304	0.02
10.4–13.4	6.1	14/231	
13.5–7.1	9.8	15/153	
17.2–71.0	12.6	16/127	

Abbreviations: HUS, hemolytic uremic syndrome; WBC, white blood cell count. ^a^ Patients with suspected HUS are included in the denominator. ^b^ All *p* values based on Fisher exact test. ^c^ Patient- (or parent/guardian) reported as having occurred at any time during course of the illness caused by O157; data was missing for some patients as follows: fever (*n* = 61), vomiting (*n* = 10), bloody stool (*n* = 17), pain and antipyretic medications (*n* = 66), antimotility drugs (*n* = 75). ^d^ During the first 10 days of illness and on or before a HUS diagnosis date; no such WBC data was available for 493 patients. For all 815 patients with data, the median time of the initial WBC was day 2 of illness (interquartile range, 1–4 days). For the 60 patients with confirmed HUS and WBC data, the median time to initial WBC was day 3 of illness (interquartile range, 1–5 days).

**Table 3:** Frequency of documented antimicrobial use during the first 7 days of illness (and before HUS diagnosis) by demographic and clinical characteristics.

Characteristic	Frequency of Documented Antimicrobial Use, %	Used Antimicrobials/Total Patients No./No.	*p* Value ^a^
All patients	31.0	406/1308	
Age in years			
<5	12.6	40/318	<0.001
5 to <15	11.5	37/323	
15 to <40	45.4	154/339	
≥40	53.4	175/328	
Sex			
Female	32.1	220/685	0.37
Male	29.7	185/622	
Race			
White	32.3	359/1113	0.09
Black	30	14/47	
Asian	30	8/27	
Other	15	6/40	
Unknown	24	19/81	
Fever ^b^			
No	31.6	231/731	0.49
Yes	29.7	153/516	
Vomiting ^b^			
No	30.9	212/686	0.86
Yes	31.4	192/612	
Bloody stool ^b^			
No	15.5	23/148	<0.001
Yes	32.7	376/1150	
Day of illness at time of first medical encounter			
1–3	34.8	291/837	<0.001
4–7	28.5	113/396	
>7	3	2/75	
Acetaminophen ^b^			<0.001
No	34.0	323/946	
Yes	20.8	62/296	
Nonsteroidal anti-inflammatory drugs ^b^			
No	32.1	341/1063	0.04
Yes	24.6	44/179	
Antimotility drugs ^b^			
No	28.3	270/955	0.001
Yes	40.3	112/278	
Highest WBC (×10^9^/L) ^c^			
3.9–10.3	34.2	104/304	0.04
10.4–13.4	42.0	97/231	
13.5–17.1	47.7	73/153	
17.2–71.0	40.9	52/127	

Abbreviations: HUS, hemolytic uremic syndrome; WBC, white blood cell count. ^a^ All *p* values based on Fisher exact test. ^b^ Patient- (or parent/guardian) reported as having occurred at any time during course of the illness caused by STEC O157 infection; data was missing for some patients as follows: fever (*n* = 61 patients), vomiting (*n* = 10), bloody stool (*n* = 17), pain and antipyretic medications (*n* = 66), antimotility drugs (*n* = 75). ^c^ WBC during the first 10 days of illness was missing for 434 patients.

**Table 4:** Frequency of antimicrobial exposure by HUS status and the association between recorded antimicrobial exposures during the first 7 days of illness and the development of confirmed HUS ^a^.

Antimicrobial Exposure	HUS ^b^			Crude Odds Ratio for HUS	95% CI	Adjusted Odds Ratio for HUS ^c^	95% CI	
	Confirmed (*n* = 102) No. (%)	Suspected (*n* = 27) No. (%)	None (*n* = 1179) No. (%)					Patients in Adjusted Model ^d^ No.
Any antimicrobial	23 (22.5)	9 (33)	374 (31.7)	0.63	0.39–1.01	1.37	0.73–2.57	1155
Fluoroquinolone	5 (4.9)	2 (7)	250 (21.2)	0.20	0.08–0.51	0.30	0.04–2.53	1024
Metronidazole	12 (11.8)	4 (15)	198 (16.8)	0.62	0.33–1.16	1.72	0.65–4.59	990
Macrolide	0 (0)	0 (0)	12 (1.0)	0.61 ^e^	0.00–2.94	^f^		827
β-lactam	10 (9.8)	4 (15)	48 (4.1)	2.12	1.03–4.36	2.80	1.14–6.89	854
SXT	8 (7.8)	2 (7)	26 (2.2)	3.13	1.18–7.42	2.37	0.85–6.60	811

Abbreviations: HUS, hemolytic uremic syndrome; CI, confidence interval; SXT, trimethoprim-sulfamethoxazole. ^a^ The 27 patients with suspected HUS were excluded from all calculations of odds ratios and confidence intervals. For antimicrobial class odds ratio calculations, patients exposed to antimicrobial classes other than the one being assessed were excluded. ^b^ Confirmed HUS was defined as including all the following abnormalities during the first 10 days of illness: (1) hemoglobin or hematocrit below age- and gender-specific thresholds [16], (2) fragmented erythrocytes on peripheral blood smear, (3) platelets <150 × 10^9^/L, and (4) serum creatinine ≥ 88.4 µmol/L if <13 years old or ≥ 132.6 µmol/L if ≥13 years old; values were assumed to be normal when any of these data were not identified through medical record abstraction. Suspected HUS was defined as an illness diagnosed by a treating clinician as HUS or thrombotic thrombocytopenic purpura in a patient with fragmented erythrocytes on peripheral blood smear, without other data available required for confirmed case classification. ^c^ All analyses adjusted for age quartile, time to healthcare presentation, and patient- (or parent/guardian) reported fever, vomiting, and acetaminophen use; the fluoroquinolone odds ratio was adjusted for documented exposure to metronidazole, and the metronidazole odds ratio was adjusted for documented exposure to fluoroquinolones. ^d^ Of the 1281 patients not excluded for suspected HUS, 126 were excluded from all adjusted models because data were missing for 1 or more covariates (acetaminophen (*n* = 64), fever (*n* = 60), vomiting (*n* = 9)). An additional variable number of patients with documented exposure to other antimicrobial classes were excluded from each antimicrobial class-specific model. ^e^ Median unbiased estimate. ^f^ Multivariable model did not converge.

**Table 5:** Frequency of antimicrobial exposure by HUS status and the association between recorded antimicrobial exposures during the first 3 days of illness and the development of confirmed HUS ^a^.

Antimicrobial Exposure	HUS ^b^			Crude Odds Ratio for HUS	95% CI	Adjusted Odds Ratio for HUS ^c^	95% CI	
	Confirmed (*n* = 102) No. (%)	Suspected (*n* = 27) No. (%)	None (*n* = 1179) No. (%)					No. Patients in Adjusted Model ^d^
Any antimicrobial	10 (9.8)	4 (14.8)	211 (17.9)	0.50	0.26–0.97	0.83	0.35–1.96	1155
Fluoroquinolone	2 (2.0)	1 (3.7)	137 (11.6)	0.15	0.04–0.63	^f^		1081
Metronidazole	5 (4.9)	3 (11.1)	112 (9.5)	0.47	0.19–1.18	0.78	0.17–3.52	1062
Macrolide	0 (0)	0 (0)	8 (0.7)	0.96 ^e^	0.00–4.85	^f^		985
β-lactam	4 (3.9)	1 (3.7)	23 (2.0)	1.83	0.62–5.41	1.86	0.44–7.84	982
TMP/SMX	5 (4.9)	1 (3.7)	11 (0.9)	4.78	1.63–14.06	2.64	0.59–11.96	968

Abbreviation: HUS, hemolytic uremic syndrome; CI, confidence interval; TMP/SMX, trimethoprim-sulfamethoxazole. ^a^ The 27 patients with suspected HUS were excluded from all calculations of odds ratios and confidence intervals. For antimicrobial class odds ratio calculations, patients exposed to antimicrobial classes other than the one being assessed were excluded. ^b^ Confirmed HUS was defined as including all of the following abnormalities during the first 10 days of illness: (1) hemoglobin or hematocrit below age- and gender-specific thresholds [16], (2) fragmented erythrocytes on peripheral blood smear, (3) platelets <150 × 10^9^/L, and (4) serum creatinine ≥ 88.4 µmol/L if <13 years old or ≥132.6 µmol/L if ≥13 years old; values were assumed to be normal when any of these data were not identified through medical record abstraction. Suspected HUS was defined as an illness diagnosed by a treating clinician as HUS or thrombotic thrombocytopenic purpura in a patient with fragmented erythrocytes on peripheral blood smear, without other data available required for confirmed case classification. ^c^ All analyses adjusted for age quartile, time to healthcare presentation, and patient- (or parent/guardian) reported fever, vomiting, and acetaminophen use; the fluoroquinolone odds ratio was adjusted for documented exposure to metronidazole, and the metronidazole odds ratio was adjusted for documented exposure to fluoroquinolones. ^d^ Of the 1281 patients not excluded for suspected HUS, 126 were excluded from all adjusted models because data were missing for 1 or more covariates (acetaminophen (*n* = 64), fever (*n* = 60), vomiting (*n* = 9)). An additional variable number of patients with documented exposure to other antimicrobial classes were excluded from each antimicrobial class-specific model. ^e^ Median unbiased estimate. ^f^ Multivariable model did not converge.

**Table 6:** Secondary models for the association between β-lactam treatment during the first 7 days of illness and development of HUS.

Category ^a^	HUS Definition ^b^	Source of Antibiotic Exposure Data ^c^	No. Patients Included	WBC Included as Model Covariate ^d^	Adjusted OR ^e^	95% CI
A	Confirmed or suspected	Documented or reported	812	No	3.90	1.80–8.43
				Yes	3.71	1.71–8.07
		Documented	874	No	3.45	1.54–7.74
				Yes	3.22	1.43–7.25
	Confirmed (suspected excluded)	Documented or reported	792	No	3.41	1.47–7.94
				Yes	3.48	1.49–8.14
		Documented	854	No ^f^	2.80	1.14–6.89
				Yes	2.83	1.15–7.01
	Confirmed (suspected considered no HUS)	Documented or reported	812	No	2.67	1.18–6.03
				Yes	2.74	1.21–6.23
		Documented	874	No	2.18	0.91–5.20
				Yes	2.23	0.93–5.34
B	Confirmed (suspected excluded)	Documented or reported	490	No	2.75	1.12–6.73
				Yes	2.94	1.18–7.28
		Documented	511	No	1.95	0.77–4.94
				Yes	2.06	0.80–5.29
	Confirmed (suspected considered no HUS)	Documented or reported	508	No	2.26	0.96–5.32
				Yes	2.41	1.01–5.77
		Documented	529	No	1.63	0.69–4.00
				Yes	1.73	0.70–4.30

Abbreviations: HUS, hemolytic uremic syndrome; WBC, white blood cell count; CI, confidence interval. ^a^ Models in category A assumed that test results for patients with missing results of any test used to define HUS would have been normal (i.e., not indicative of HUS) had they been performed. Models in category B excluded any patient missing data for any test used to define HUS (creatinine, platelets, hemoglobin or hematocrit, or peripheral blood smear). ^b^ Confirmed HUS was defined as including all of the following abnormalities during the first 10 days of illness: (1) hemoglobin or hematocrit below age- and gender-specific thresholds, (2) fragmented erythrocytes on peripheral blood smear, (3) platelets < 150 × 10^9^/L, and (4) serum creatinine ≥ 88.4 µmol/L if <13 years old or ≥132.6 µmol/L if ≥13 years old; Suspected HUS was defined as illness diagnosed by a treating clinician as HUS or thrombotic thrombocytopenic purpura in a patient with fragmented erythrocytes on peripheral blood smear, but lacking complete laboratory documentation required for a confirmed case. ^c^ Documented exposure was defined as identification of antibiotic administration or prescription in medical records; reported exposure was defined as patient- (or legal guardian) reported exposure. ^d^ In models that included the initial WBC value during the first 10 days of illness (and before HUS diagnosis) as a covariate, a dichotomous variable was used (WBC ≥ 17.2 × 10^9^/L versus <17.2 × 10^9^/L); patients with no WBC count documented were assumed to have WBC <17.2 × 10^9^/L. ^e^ All models adjusted for age quartile, time to healthcare presentation, and patient- (or parent/guardian) reported fever, vomiting, and acetaminophen use. ^f^ This is the primary model and these modelling assumptions were used for Table 3.

## Data Availability

The data is available by making a data request to FoodNet (Foodnet@cdc.gov) or the corresponding author, with subsequent approval of the FoodNet Steering Committee. Due to the inclusion of personally identifiable information, a complete dataset is not publicly available.

## Supplementary Materials

The following are available online at https://www.mdpi.com/article/10.3390/microorganisms9091997/s1, Table S1: Demographic and clinical characteristics of study participants eligible for inclusion in the primary multivariable adjusted models and participants excluded due to missing data, Table S2: Secondary models for the association between trimethoprim-sulfamethoxazole treatment during the first 7 days of illness and development of HUS, Table S3: Secondary models for the association between metronidazole treatment during the first 7 days of illness and development of HUS. Table S4: Secondary models for the association between fluoroquinolone treatment during the first 7 days of illness and development of HUS, Study Questionnaire 1: Main Questionnaire, Study Questionnaire 2: Medical Provider Follow-up Questionnaire, Study Questionnaire 3: Hospital Follow-up Questionnaire, Study Questionnaire 4: Antibiotic Follow-up Questionnaire.
