# Supplementary material for: Risk of Hemolytic Uremic Syndrome Related to Treatment of Escherichia coli O157 Infection with Different Antimicrobial Classes
Source: Microorganisms. 2021 Sep 21;9(9):1997. doi: 10.3390/microorganisms9091997 (PMC8466573; doi:10.3390/microorganisms9091997)
Supplement: Supplementary file 1 [file microorganisms-09-01997-s001.zip › Antibiotic Follow-up Form.pdf]

E. coli O157 COHORT STUDY ANTIBIOTIC FOLLOW-UP

COMPLETE ANTIBIOTIC FOLLOWUP FORM FOR ANTIBIOTIC EXPOSURES LISTED IN Q 12 AND 13 IF THE CASE-PATIENT CANNOT REMEMBER THE NAME OF THE ANTIBIOTIC OR START DATE

COHORT Study ID [COHORT_ID] __ __ __ __ __ **(Enter 2 letter state code and number, eg XX001)**

State Laboratory Isolate ID Number* _______[SLABS_ID]_________________ *This is required

Illness onset date _______[ILL_ONSET]_____ Person Completing Form ____[ABX_COMP]___

FILL IN FROM COHORT QUESTIONNAIRE, CIRCLE QUESTION→

[**Antibiotics 2 - 9, same as listed here with first 5 characters of each variable (ABX1_) changed to indicate antibiotic 'ABX2_' through 'ABX9_'**]

Question: [ABX1_Y] **12** (1) **OR 13** (2)
Date prescribed: [ABX1_PER]
Physician name: ~~[ABX1_PHY]~~
Clinic/ER name: ~~[ABX1_CLN]~~
Address: ~~[ABX1_ADD]~~
City, State: [ABX1_CTY], [ABX1_ST]
Phone: ~~[ABX1_PHO]~~

"I am calling from the _____________ State Health Department, we are conducting a study with the Centers for Disease Control among persons infected with E. coli O157. We recently enrolled ________________ ***(CASE NAME)*** *in the study. We are contacting medical providers to confirm information regarding antibiotic use reported during the interview. ________________* ***(CASE NAME)*** *indicated that he/she took antibiotics that were prescribed or given by your office but could not recall some details about the antibiotics. We would like to confirm the name of the antibiotic and the date it was given or prescribed. "*

________________ **(CASE NAME)** indicated that [he / she] took an antibiotic that was prescribed or given __________(**DATE PRESCRIBED).**

1. Was an antibiotic prescribed or given on __________**(DATE PRESCRIBED) or in the three weeks before__________(Illness onset date)?** [ABX1_PRE]

Yes.. 1
No..**END**……….. 2
Don't know/Not sure............**END**.......…… 7

IF YES → Please answer the following, what is the:
A. Name of antibiotic **(ENTER ANTIBIOTIC NAME)**
B. Start date of antibiotic prescription or date given **(ENTER mm/dd/yy)**
C. Number of days of treatment given or prescribed (e.g. 10 days, 7 days etc.)

A. Antibiotic Name	B. Start date? (999999 if unknown)	D. Number of days
[ABX1_NM]<br>(XX=ABX CODE SHEET)	[ABX1_DT]	[ABX1_DY]

2. Why was this antibiotic given or prescribed? [ABX1_WHY]

Ear, sinus, upper respiratory infection……….. 1
Bronchitis or pneumonia……………………… 2
Urinary tract infection………………………... 3
Skin infection………………………………….... 4
Diarrhea………………………………………… 5
Other…………………………….. 6
SPECIFY ____[ABX1_SPEC]___
Don't Know……………………… 7
Refused………………………….. 9

Thank you for your help with this study. **(END)**

[INTERVIEWER ONLY] → Were you able to obtain information from this medical provider about the antibiotic that was taken?

[ABX1_INT] Yes.................... 1 No..…......... 2
→IF NO, why not? [ABX1_NO]
Physician information incorrect or insufficient... 1
Physician/Office not able to find records........... 2
Physician/Office refused to provide information.....3
Other……………………………………….…………4
SPECIFY______[ABX1_NO_SPEC]_________
