# Supplementary material for: Risk of Hemolytic Uremic Syndrome Related to Treatment of Escherichia coli O157 Infection with Different Antimicrobial Classes
Source: Microorganisms. 2021 Sep 21;9(9):1997. doi: 10.3390/microorganisms9091997 (PMC8466573; doi:10.3390/microorganisms9091997)
Supplement: Supplementary file 1 [file microorganisms-09-01997-s001.zip › Hospital Form.pdf]

E. coli O157 COHORT STUDY: HOSPITAL FOLLOW-UP

COMPLETE "HOSPITAL FOLLOW-UP" FORM FOR EACH HOSPITAL LISTED, CONTACT AT LEAST 3 WEEKS AFTER ILLNESS ONSET DATE

COHORT Study ID [COHORT_ID] __ __ __ __ __ **(2 letter state code and number, eg XX001)**

State Laboratory Isolate ID Number* ________[SLABS_ID]______ *This is required

Illness onset date _____[ILL_ONSET]_______

[**Change "HOSP1" TO "HOSP2", "HOSP3" or "HOSP4" in variables below for hospitals 2, 3, AND 4, respectively**]
Hospital **1** **2** **3** **4** [HOSP]
Hospital admission date ___[HOSP1_DT] __ (mm/dd/yy)
Hospital Name/CODE: ______~~[HSP1_NM]~~______________ **Address:** ______~~[HSP1_ADD]~~ ________
City, State: ______[HOSP1_CTY], [HOSP1_ST] __ **Phone:** ______~~[HSP1_PHO]~~ _________

[**ADD "_2", "_3", OR "_4 to end of ALL variables below for hospitals 2, 3, or 4, respectively**]
(COMPLETE AFTER CONTACTING HOSPITAL) Were you able to complete a "Hospital Follow-Up" form for this hospital?
[HOSP_COM] Yes.................................. 1 **IF YES**, Date completed __[HOSP_COM_DT]__ (mm/dd/yy)
No................................... 2

→→**IF NO,** why were you not able to complete a "Hospital Follow-Up" form? [HOSP_COM_RE]
Hospital information incorrect........................... 1
Hospital refused access to records..................... 2
Records at hospital unavailable......................... 3, **SPECIFY**____[HOSP_COM_SPEC3]_____
Other.. 4, **SPECIFY**____[HOSP_COM_SPEC4]_____

---

1. Was the patient admitted to this hospital more than once for this illness? [HOSP_ADM]
Yes................... 1 Don't know/Not sure.......... 7
No.................... 2

→ **IF YES**, how many times was the patient admitted? [HOSP_ADM_TI]__ __, Don't know/Not sure.......... 77

2. What was the first date of admission to this hospital for this illness? _[HOSP_ADM_DT]_
mo day yr
Don't know/Not 777777

3A. Blood type [HOSP_BLD]
A…………………………1 O………………………………….. 4
B…………………………2 Don't know/Not sure...................... 7
AB……………………… 3

3B. Height: _[HOSP_HT] ft/in OR cm (circle), Date (closest to illness onset): __[HOSP_HT_DT]__ DK/Not sure.....777777

Weight(admit): [HOSP_WT]lbs OR kg (circle),Date (closest to illness onset): __[HOSP_WT_DT]__ DK/Not sure.....777777

4. A. What date was stool submitted for testing / culture at this hospital? _[HOSP_CUL_DT]_,
Don't know/Not sure... 777777 mo day yr
Not done…**GO TO 5**…………..999999

B. Was Shiga toxin testing performed? [HOSP_SHIG] Yes...........1 No...........2 Don't know/Not sure......... 7
→**IF YES,** →What was the result? [SHIG_RES]
Positive…1 Negative…………2 Don't know/Not sure.......... 7

→What date were the results reported? __[SHIG_DT]___
mo day yr
Don't know/Not sure.......... 777777

C. What date was *E. coli* O157 isolation reported from the lab? __[HOSP_ECOLI]__
mo day yr
Don't know/Not sure.......... 777777

5. Was a diagnosis of hemolytic uremic syndrome or HUS documented in the 3 weeks after the illness onset date?
[HOSP_HUS] Yes.................... 1 Don't know/Not sure.......... 7
No..................... 2
→ **IF YES**, on what date was HUS diagnosed? __[HUS_DT]__
mo day yr
Don't know/Not ……........ 777777

6. Was a diagnosis of thrombotic thrombocytopenic purpura or TTP documented in the 3 weeks after the illness onset date?
[HOSP_DDT] Yes.................... 1 Don't know/Not sure.......... 7
No..................... 2
→ **IF YES**, on what date was TTP diagnosed? ____[TTP_DT]____
mo day yr
Don't know/Not 777777

7. Was the patient hospitalized in an intensive care unit? [HOSP_ICU]

Yes..	1
No..**GO TO Q9** …..	2
Don't know/Not sure.................**GO TO Q9**	7

8. How many nights were spent in ICU in the 3 weeks after the illness onset date? [ICU_NITE] __ __ nights

Still in ICU…………………………………………………………………	77
Don't know/Not sure..	99

9. What was the date of discharge from this hospital? __[HOSP_DIS]__
mo day yr
Still in Hospital....................**GO TO 11**...... 777777
Don't know/Not sure…................................. 999999

10. Where did the patient go after discharge from the hospital? [DIS_GO]

Home…………………………………………………………………	1
Transferred to Another Hospital………………………………………………	2
Assisted Living Facility (e.g. board and care)……………………………..	3
Skilled-nursing facility……………………………………………………..	4
Deceased……………………………………………………………………	5
Other…..……………………………………………………………………	6
**SPECIFY** __[DIS_SPEC]________________________________	
Don't Know…………………………………………………………………..	7

→**IF "DECEASED"** What was listed as the cause(s) of death?
________[DIS_CAUSE]_________

11. Was the patient treated with antibiotics or prescribed antibiotics at this hospital for this illness? [HOSP_ABX]

Yes............................. **IF YES, Complete 12**...	1
No...	2
Don't know/Not sure...	7

12. Antibiotic worksheet- **(COMPLETE ROW FOR EACH ANTIBIOTIC DOCUMENTED IN THE CHART AT THIS HOSPITAL)**

A. Antibiotic names and codes **(USE ANTIBIOTIC CODES IN ADMIN PACKET)**
B. Was the antibiotic administered intravenously?
C. Enter date antibiotic was first administered **(ENTER mmddyy, Unk=777777)**
D. Enter number of days received **(ENTER 77 if Unk or discharge med)**
E. Were antibiotics prescribed at discharge?
F. What is the start date of the prescription? **(ENTER discharge date when the Ab was prescribed if no start date is specified, Unk=777777)**
G. How many days were antibiotics prescribed (eg 10 days, 14 days)? **(ENTER 77 if Unk)**
H. Was this antibiotic discontinued **because** of the diagnosis of *E. coli* O157?

**A.Antibiotic Name**	**Code**	**B. IV** Yes(1) No(2) DK(7)	**C. Date mmddyy**	**D. # of Days**	**E. Prescribed at discharge?** Yes(1) No(2) DK(7)	**F. Start date of prescription**	**G. # Days prescribed**	**H. Discontinued because of E. coli O157 diagnosis?** Yes(1) No(2) DK(7)
	[HOSP_ABX1]	[HOSP_ABX1_IV]	[HOSP_ABX1_ST]	[HOSP_ABX1_NUM]	[HOSP_ABX1_DIS]	[HOSP_ABX1_PER]	[HOSP_ABX1_DYS]	[HOSP_ABX1_DIAG]
	[HOSP_ABX2]	[HOSP_ABX2_IV]	[HOSP_ABX2_ST]	[HOSP_ABX2_NUM]	[HOSP_ABX2_DIS]	[HOSP_ABX2_PER]	[HOSP_ABX2_DYS]	[HOSP_ABX2_DIAG]
	[HOSP_ABX3]	[HOSP_ABX3_IV]	[HOSP_ABX3_ST]	[HOSP_ABX3_NUM]	[HOSP_ABX3_DIS]	[HOSP_ABX3_PER]	[HOSP_ABX3_DYS]	[HOSP_ABX3_DIAG]
**OTHER – SPECIFY** [HOSP_OTH1_NM]	[HOSP_OTH1] = (52)	[HOSP_OTH1_IV]	[HOSP_OTH1_ST]	[HOSP_ OTH1_NUM]	[HOSP_ OTH1_DIS]	[HOSP_ OTH1_PER]	[HOSP_ OTH1_DYS]	[HOSP_ OTH1_DIAG]
**OTHER – SPECIFY** [HOSP_OTH2_NM]	[HOSP_OTH1] = (53)	[HOSP_OTH2_IV]	[HOSP_OTH2_ST]	[HOSP_ OTH2_NUM]	[HOSP_ OTH2_DIS]	[HOSP_ OTH2_PER]	[HOSP_ OTH2_DYS]	[HOSP_ OTH2_DIAG]

13. Use the following charts to record symptoms, laboratory values, and treatment variables that are documented in medical records from this hospital during the first 10 days of this illness. **RECORD DAYS THAT THE CASE WAS HOSPITALIZED, LEAVE ENTIRE COLUMN BLANK IF CASE WAS NOT HOSPITLALIZED ON THAT DAY; PLEASE NOTE THE DATE THAT LABORATORY VALUES REFER TO, RECORD ON THE APPROPRIATE DATE.**

13A. REPEAT FOR EACH DAY THAT THE CASE WAS HOSPITALIZED DURING THE FIRST 10 DAYS OF THIS ILLNESS, FILL IN DATES THAT CASE WAS HOSPITALIZED Y (1) N (2) DK (7)

A1. Was a temperature recorded? → **If YES, A2**. What was the highest temperature recorded that day? (Enter 777.7=DK, 999.9=Refused)
B. Vomiting documented?
C. Diarrhea documented?
D. Blood (visible or occult) in stool documented?

**DATE**	**D1 ONSET** [HOSP_DY1]	**DAY2** [HOSP_DY2]	**DAY3** [HOSP_DY3]	**DAY4** [HOSP_DY4]	**DAY5** [HOSP_DY5]	**DAY6** [HOSP_DY6]	**DAY7** [HOSP_DY7]	**DAY8** [HOSP_DY8]	**DAY9** [HOSP_DY9]	**DAY10** [HOSP_DY10]
**A1. Temp**	[DY1_TEMP]	[DY2_TEMP]	[DY3_TEMP]	[DY4_TEMP]	[DY5_TEMP]	[DY6_TEMP]	[DY7_TEMP]	[DY8_TEMP]	[DY9_TEMP]	[DY10_TEMP]
**A2. High T**	[DY1_HTEMP]	[DY2_HTEMP]	[DY3_HTEMP]	[DY4_HTEMP]	[DY5_HTEMP]	[DY6_HTEMP]	[DY7_HTEMP]	[DY8_HTEMP]	[DY9_HTEMP]	[DY10_HTEMP]
**B. Vomit**	[DY1_VOMIT]	[DY2_VOMIT]	[DY3_VOMIT]	[DY4_VOMIT]	[DY5_VOMIT]	[DY6_VOMIT]	[DY7_VOMIT]	[DY8_VOMIT]	[DY9_VOMIT]	[DY10_VOMIT]
**C. Diar**	[DY1_DA]	[DY2_DA]	[DY3_DA]	[DY4_DA]	[DY5_DA]	[DY6_DA]	[DY7_DA]	[DY8_DA]	[DY9_DA]	[DY10_DA]
**D. Blood**	[DY1_BLOOD]	[DY2_ BLOOD]	[DY3_ BLOOD]	[DY4_ BLOOD]	[DY5_ BLOOD]	[DY6_ BLOOD]	[DY7_ BLOOD]	[DY8_ BLOOD]	[DY9_ BLOOD]	[DY10_ BLOOD]

13B. RECORD DAYS THAT THE CASE WAS HOSPITALIZED, LEAVE ENTIRE COLUMN BLANK IF CASE WAS NOT HOSPITLALIZED ON THAT DAY, REPEAT FOR EACH DAY THAT CASE WAS HOSPITALIZED DURING THE FIRST 10 DAYS OF THIS ILLNESS

A. Highest BUN
B. Highest Creatinine
C. Highest LDH
D. Highest Amylase
E. Highest Lipase
F. Highest Bilirubin
G. Highest Transaminase Alanine (ALT)
H. Highest Transaminase Aspartate (AST)
I. Highest WBC count (per mm^3)
J. Lowest Hematocrit (%)
K. Lowest Hemoglobin
L. Lowest Platelet count (per mm^3)
M. Blood smear performed, **IF yes,** M1., Were erythrocyte changes / fragments noted, **If yes**, Specify (eg schistocytes, burr cells, helmet cells)
N. Urinalysis performed **IF yes**, N1., Hemanaturia, **If yes**, Specify (eg 1+, 2+, 3+, 4+) N2., Proteinuria, **If yes**, Specify (eg 1+, 2+, 3+)

**DATE**	**D1 ONSET** [HOSP_DY1]	**HIGHEST VALUE**	**LOWEST VALUE**
**Labs**	Y (1) N (2) [DY1_LABS]		
**A. High BUN**	[DY1_BUN] mg/dL	*DATE* [DT_BUN] [DY2_BUN] mg/dL	
**B. High Cr**	[DY1_CR] mg/dL	*DATE* [DT_CR] [DY2_CR] mg/dL	
**C. High LDH**	[DY1_LDH] U/L	*DATE* [DT_LDH] [DY2_LDH] U/L	
**D. High Bilirubin**	[DY1_BIL] mg/dL	*DATE* [DT_BIL] [DY2_BIL] mg/dL	
**E. Highest WBC**	[DY1_WBC]	*DATE* [DT_WBC] [DY2_WBC]	
**F. Lowest HCT**	[DY1_HCT]		*DATE* [DT_HCT] [DY2_BUN] mg/dL
**G. Lowest HB**	[DY1_HEMO]		*DATE* [DT_HEMO] [DY2_HEMO] mg/dL
**H. Lowest PLT**	[DY1_HPLAT]		*DATE* [DT_HPLAT] [DY2_HPLAT] mg/dL
**I. Blood smear** (Anytime during illness)	[DY1_BLOOD_SM] Y(1) N(2) DK(7) *DATE* [DT_BLOOD_SM]		
**I1 RBC frags**	[DY1_RBC] Y(1) N(2) DK(7)		
**I2 RBC specify**	[DY1_RBC_SPEC]		
**J. Urinalysis** (Anytime during illness)	[DY1_URINE] Y(1) N(2) DK(7)		
**J1 Hematuria**	[DY1_HEMAT] Y(1) N(2) DK(7) *DATE* [DT_HEMAT]		
**J2 Proteinuria**	[DY1_PROT] Y(1) N(2) DK(7) *DATE* [DT_PROT]		

13C. RECORD DAYS THAT THE CASE WAS HOSPITALIZED, LEAVE ENTIRE COLUMN BLANK IF CASE WAS NOT HOSPITLALIZED ON THAT DAY REPEAT FOR EACH DAY THAT CASE WAS HOSPITALIZED

A. Were IV fluids administered Y(1) N(2) DK(7)

If Yes, **A1.** Type of fluids: Normal saline……1 ½ Normal saline….2 D5W……….3 Other……….4, **If other, specify other fluid type**

A2. Volume (Liters / day)

B. Were blood products given: Y(1) N(2) DK(7) **If Yes**, Which products: B1. Packed cells B2. Whole blood B3. Platelets B4. Plasma

C Were any anti-diarrheal medications administered (lomotil (diphenoxylate), loperamide, bismuth subsalicylate, kaolin pectin) Y(1) N(2) DK(7)

If Yes, C1. Biologic agents: probiotics

C2. Anti-motility agents: lomotil (diphenoxylate), loperamide

C3. Adsorbent agents: bismuth subsalicylate, kaolin pectin

D. Other anti-diarrheal agents, Y(1) N(2) DK(7) **If Yes**, Specify

DATE	**D1 ONSET** [HOSP_DY1]	**DAY2** [HOSP_DY2]	**DAY3** [HOSP_DY3]	**DAY4** [HOSP_DY4]	**DAY5** [HOSP_DY5]	**DAY6** [HOSP_DY6]	**DAY7** [HOSP_DY7]	**DAY8** [HOSP_DY8]	**DAY9** [HOSP_DY9]	**DAY 10 +** [HOSP_DY10]
**A. IV fluids**	[DY1_IV]	[DY2_IV]	[DY3_IV]	[DY4_IV]	[DY5_IV]	[DY6_IV]	[DY7_IV]	[DY8_IV]	[DY9_IV]	[DY10_IV]
**A1. Type of fluids**	[DY1_IV_TY]	[DY2_IV_TY]	[DY3_IV_TY]	[DY4_IV_TY]	[DY5_IV_TY]	[DY6_IV_TY]	[DY7_IV_TY]	[DY8_IV_TY]	[DY9_IV_TY]	[DY10_IV_TY]
**Specify other fluids**	[DY1_IV_OTH]	[DY2_IV_OTH]	[DY3_IV_OTH]	[DY4_IV_OTH]	[DY5_IV_OTH]	[DY6_IV_OTH]	[DY7_IV_OTH]	[DY8_IV_OTH]	[DY9_IV_OTH]	[DY10_IV_OTH]
**A2. Volume (Liters/day)**	[DY1_IV_VOL]	[DY2_IV_VOL]	[DY3_IV_VOL]	[DY4_IV_VOL]	[DY5_IV_VOL]	[DY6_IV_VOL]	[DY7_IV_VOL]	[DY8_IV_VOL]	[DY9_IV_VOL]	[DY10_IV_VOL]
**B. Blood products**	[DY1_BLD_PR]	[DY2_BLD_PR]	[DY3_BLD_PR]	[DY4_BLD_PR]	[DY5_BLD_PR]	[DY6_BLD_PR]	[DY7_ BLD_PR]	[DY8_BLD_PR]	[DY9_BLD_PR]	[DY10_BLD_PR]
**B1. P cells**	[DY1_PCELLS]	[DY2_PCELLS]	[DY3_PCELLS]	[DY4_PCELLS]	[DY5_PCELLS]	[DY6_PCELLS]	[DY7_PCELLS]	[DY8_PCELLS]	[DY9_PCELLS]	[DY10_ PCELLS]
**B2. W blood**	[DY1_WCELLS]	[DY2_WCELLS]	[DY3_WCELLS]	[DY4_WCELLS]	[DY5_WCELLS]	[DY6_WCELLS]	[DY7_WCELLS]	[DY8_WCELLS]	[DY9_WCELLS]	[DY10_WCELLS]
**B3. Platelets**	[DY1_PLAT]	[DY2_PLAT]	[DY3_PLAT]	[DY4_PLAT]	[DY5_PLAT]	[DY6_PLAT]	[DY7_PLAT]	[DY8_PLAT]	[DY9_PLAT]	[DY10_PLAT]
**B4. Plasma**	[DY1_PLASMA]	[DY2_PLASMA]	[DY3_PLASMA]	[DY4_PLASMA]	[DY5 PLASMA]	[DY6_PLASMA]	[DY7_PLASMA]	[DY8_ PLASMA]	[DY9_ PLASMA]	[DY10_ PLASMA]
**C. Anti-diarrheal**	[DY1_ANTIDA]	[DY2_ANTIDA]	[DY3_ANTIDA]	[DY4_ANTIDA]	[DY5_ANTIDA]	[DY6_ANTIDA]	[DY7_ANTIDA]	[DY8_ANTIDA]	[DY9_ANTIDA]	[DY10_ANTIDA]
**C1. Probiotic**	[DY1_PROB]	[DY2_PROB]	[DY3_PROB]	[DY4_PROB]	[DY5_PROB]	[DY6_PROB]	[DY7_PROB]	[DY8_PROB]	[DY9_PROB]	[DY10_PROB]
**C2. Anti-mot**	[DY1_MOT]	[DY2_MOT]	[DY3_MOT]	[DY4_MOT]	[DY5_MOT]	[DY6_ MOT]	[DY7_MOT]	[DY8_MOT]	[DY9_MOT]	[DY10_ MOT]
**C3. Adsorb**	[DY1_ADSORB]	[DY2_ADSORB]	[DY3_ADSORB]	[DY4_ADSORB]	[DY5_ADSORB]	[DY6_ ADSORB]	[DY7_ADSORB]	[DY8_ADSORB]	[DY9_ADSORB]	[DY10_ ADSORB]
**D. Other anti-diarr**	[DY1_OTHER _ANTIDA]	[DY2_OTHER _ANTIDA]	[DY3_OTHER _ANTIDA]	[DY4_OTHER _ANTIDA]	[DY5_OTHER _ANTIDA]	[DY6_OTHER _ANTIDA]	[DY7_OTHER _ANTIDA]	[DY8_OTHER _ANTIDA]	[DY9_OTHER _ANTIDA]	[DY10_OTHER _ANTIDA]
**D1. Specify**	[DY1_OTHER _SPEC]	[DY2_OTHER _SPEC]	[DY3_OTHER _SPEC]	[DY4_OTHER _SPEC]	[DY5_OTHER _SPEC]	[DY6_OTHER _SPEC]	[DY7_OTHER _SPEC]	[DY8_OTHER _SPEC]	[DY9_OTHER _SPEC]	[DY10_OTHER _SPEC]
