# Supplementary material for: Risk of Hemolytic Uremic Syndrome Related to Treatment of Escherichia coli O157 Infection with Different Antimicrobial Classes
Source: Microorganisms. 2021 Sep 21;9(9):1997. doi: 10.3390/microorganisms9091997 (PMC8466573; doi:10.3390/microorganisms9091997)
Supplement: Supplementary file 1 [file microorganisms-09-01997-s001.zip › Main Form.pdf]

E. coli **O157** COHORT QUESTIONNAIRE

<u>SECTION A</u> **COHORT Study ID** __ __ __ __ __ [COHORT_ID] (All cases / all sites, enter 2 letter state code and number, eg XX001)

State Laboratory Isolate ID Number* ____________________________ *This is required [SLABS_ID]

Specimen collection date ___/ ____ /________ → Write in on calendar [SPEC_DT]

PHLIS ID _________________ [PHLIS_ID] (Sites reporting through PHLIS)
Other ID _________________ [OTH_ID] (Specify: ___________________________________) [OTH_ID_DES]

- **INTERVIEWER INSTRUCTIONS IN BOLD**
- **BEFORE CONTACTING CASE, HAVE "ADMIN PACKET" & CALENDAR, MARK SPECIMEN COLL DATE**

<u>SECTION B-1</u>: *"Let's begin. Because I will be asking about specific dates around the time of [your / your child's] illness, it may be helpful for you to have a calendar or day planner in front of you. Would you like to get one before we start?"*

1. Have you or another family member already spoken with someone else from the health department about this illness? [HD_CON]

   Yes.................... 1 Don't know/Not sure.......... 7
   No..................... 2 Refused.............................. 9

   →**IF YES, "***Even though some of the questions may be similar to ones you have been asked before, we need to ask them in the same way to each person who had this infection.***" (GO TO 2)**
   →**IF NO,** *"Someone else from the health department may contact you at a later time."* **(GO TO 2)**

2. What county [do you / does your child] reside in? _________________________ [COUNTY]

   Don't know/Not sure..7 7 7 7 7
   Refused...9 9 9 9 9

3. What is the date of birth of the person who was ill? _____/_____/_____ [BIRTH_DT]
   mo day yr

4. What is [your / your child's] blood type? [BLD_TPE]

   A……………………………1 O……………………………….. 4
   B……………………………2 Don't know/Not sure....................... 7
   AB……………………… 3 Refused...9

5. A. Did [you / your child] have diarrhea on the day that the *E coli* O157 infection began? →**IF YES, go to 6** [DA_DAY1]

   Yes.................... 1 Don't know/Not sure.......... 7
   No..................... 2 Refused.............................. 9

   B. If you did **not** have diarrhea on the day that the *E coli* O157 infection began, which of the following symptoms appeared first? [SYM_FST]
   (**READ CHOICES IN FIRST COLUMN, CHOOSE ONLY ONE**)

   Fever ……………………..................1
   Vomiting………............................ 2 **Don't know/Not sure......................7** **Do not read**
   Nausea…………………………… 3 **Refused... 9** **Do not read**
   Other, specify_________________ 5 [SYM_OTH]

   On what date did this symptom begin? ___/_____/_____ **(This is the ONSET DATE IF NO DIARRHEA)** [SYM_DT]
   mo day yr

6. A. Did [you / your child] have **bloody** diarrhea on the day that the *E coli* O157 infection began? [BLOODY_DY]

   Yes.................... 1 Don't know/Not sure.......... 7
   No..................... 2 Refused.............................. 9

B. On what date did [your / your child's] diarrhea begin? **(Prompt recall with specimen collection date, TRY TO FACILITATE RESPONSE)** _____/_____/_____ **(This is the ONSET DATE - write date on calendar)** [DA_DT]
mo day yr

Don't know/Not sure... 777777
Refused.. 999999

7. Do you know why [your / your child's] stool sample was tested? [REASON]
(**READ CHOICES, CHOOSE ONLY ONE**)

Ill with diarrhea…………………………………	1	Other reason………………………	5	
Ill with symptoms other than diarrhea.......………...	2	→**IF Other**, Specify______________[REASON_OTH]		
Not ill, part of *E. coli* O157 investigation...............…	3	**Don't know/Not sure......................**	**7**	**Do not read**
Suspicion of kidney problems, also called HUS……	4	**Refused..**	**9**	**Do not read**

*"I would now like to ask some additional questions about [your / your child's] symptoms during this illness. It may be difficult to remember some of the details, please answer as best you can. I'll begin by asking you about a list of symptoms that [you / your child] may have had. As a reminder you indicated that the first day of diarrhea [or other symptom] was __ /__ /_____ **(ONSET DATE),** that was a ____________ **(DAY OF WEEK).**"*

8A. Did [you/your child] have**……** **(A-E)** **…..**during this illness**?**

A.	Diarrhea?	**IF Yes**, What day did it start?,	What was the last day [you/your child] had this symptom?
B.	Fever?	**IF Yes**, What day did it start?,	What was the last day [you/your child] had this symptom?
C.	Nausea?	**IF Yes**, What day did it start?,	What was the last day [you/your child] had this symptom?
D.	Vomiting?	**IF, Yes**, What day did it start?,	What was the last day [you/your child] had this symptom?
E.	Bloody stool?	**IF Yes**, What day did it start?,	What was the last day [you/your child] had this symptom?

Did [you/your child] have…… (A-E) …..during this illness?	**Y=yes, N=no, DK= don't know / not sure, RF=refused**	**Date of symptom start Enter 99/99/99=DK**	**Date of symptom stop Enter 77/77/77 if still having, Enter 99/99/99=DK**
**A. Diarrhea**	Y N DK RF [DA]	__ /__ /___ [DA_ST]	__ /__ /___ [DA_STP]
**B. Fever**	Y N DK RF [TMP]	__ /__ /___ [TMP_ST]	__ /__ /___ [TMP_STP]
**C. Nausea**	Y N DK RF [NAUS]	__ /__ /___ [NAUS_ST]	__ /__ /___ [NAUS_STP]
**D. Vomiting**	Y N DK RF [VOM]	__ /__ /___ [VOM_ST]	__ /__ /___ [VOM_STP]
**E. Bloody stool**	Y N DK RF [BLOODY]	__/__/___ [BLOODY_ST]	__ /__ /___ [BLOODY_STP]

8B. THE FOLLOWING REFER TO THE FIRST 3 DAYS OF ILLNESS (DAY 1= ONSET DATE)

"I would now like to ask you some detailed questions about the first 3 days of [your / your child's] illness…"

A. During the first 24 hours of [your / your child's] illness, what was the highest temperature recorded?. ……..What was the highest temperature recorded on the second day of this illness? ………………What was the highest temperature on the third day of this illness? (Enter 555.5=Not recorded, Enter 777.7=DK, 999.9=Refused, circle F or C)
B. During the first 24 hours of [your / your child's] illness, how many times did [you / your child] vomit? ………………….How many times did [you / your child] vomit on the second day of this illness? ………How many times did [you / your child] vomit on the third day of this illness? (Enter 777=DK, 999=Refused)
C. During the first 24 hours of [your / your child's] illness, how many times did [you / your child] have diarrhea? …………How many times did [you / your child] have diarrhea on the second day of this illness? ………How many times did [you / your child] have diarrhea on the third day of this illness? (Enter 777=DK, 999=Refused)
D. During the first 24 hours of [your / your child's] illness, how many bloody stools did [you / your child] pass? …………How many bloody stools did [you / your child] pass on the second day of this illness? ………How many bloody stools did [you / your child] pass on the third day of this illness? (Enter 777=DK, 999=Refused)

	**DAY1 (ONSET DATE)** __/__/___ [DT1]	**DAY2** __/__/___ [DT2]	**DAY3** __/__/___ [DT3]
**A. High Temp**	_ _ _ ._ F or C [TMP_DT1]	_ _ _ ._ F or C [TMP_DT2]	_ _ _ ._ F or C [TMP_DT3]
**B. vomit number**	__ __ __ [VOM_DT1]	__ __ __ [VOM_DT2]	__ __ __ [VOM_DT3]
**C. diarrhea number**	__ __ __ [DA_DT1]	__ __ __ [DA_DT2]	__ __ __ [DA_DT3]
**D. bloody stools**	__ __ __ [BLOODY_DT1]	__ __ __ [BLOODY_DT2]	__ __ __ [BLOODY_DT3]

SECTION B-2: "*Now I would like to ask you some questions about [your/your child's] health. We would like to know about any long-standing medical conditions or other specific medical conditions in the three weeks before diarrhea [or other symptom] began, which is from __/__/_____ (3 weeks before) to __/__/_____ (ONSET DATE).*" **(Note: If onset date was not recalled, use specimen collection date)**

9. Prior to **diarrhea [or other symptom]** did [you / your child] have any of the following medical conditions? **(PLEASE READ EACH CONDITION AND RECORD YES / NO / DK / REFUSE)**

		Yes	No	DK	Refuse
**A**	Urinary tract infection during the 3 weeks before illness began [HX_UTI]	1	2	7	9
**B**	Respiratory tract infection, cold, or ear infection during the 3 weeks before illness began [HX_RTI]	1	2	7	9
**C**	Pregnancy **(FEMALE)** during the 3 weeks before illness began [HX_PRG]	1	2	7	9
**D**	Diabetes [HX_DIA]	1	2	7	9
**E**	Kidney Disease [HX_KID]	1	2	7	9
	→**IF YES**, Were you/your child on dialysis or awaiting dialysis? [HX_DIAL]	1	2	7	9
**F**	Ever had an organ or bone marrow transplant [HX_TRANS]	1	2	7	9
**G**	Chronic or long-term diarrhea [HX_DA]	1	2	7	9
**H**	Leukemia or Cancer other than skin cancer [HX_LEUK]	1	2	7	9
~~**I**~~	~~HIV / AIDS [HX_HIV]~~	~~1~~	~~2~~	~~7~~	~~9~~
**J**	Asthma [HX_ASTH]	1	2	7	9
**K**	Any other long-standing medical condition [HX_MED]	1	2	7	9
	→**IF YES**, Specify: [HX_SPEC]				

"The following questions are about medications that [you / your child] may have been taking in the **3 weeks before** [your / your child's] **diarrhea [or other symptom]** began, which is from (DAY OF WEEK) __/__/_____ (**3 weeks before**) to (DAY OF WEEK) __/__/_____ (**ONSET DATE**); Medicine bottles or records may help you remember about specific medications. Would you like to gather this information before we go on?" **(Note: If onset date was not recalled, use specimen collection date)**

10. In the **3 weeks** before [your / your child's] **diarrhea [or other symptom]** began, did [you / your child] receive any of the following types of treatments or take any of the following types of medications?

**(PLEASE READ EACH MEDICATION/TREATMENT)**		Yes	No	DK	Refuse
**A**	Any steroid inhaler, such as prednisone [INH]	1	2	7	9
**B**	Any steroid pills, such as prednisone [PILL]	1	2	7	9
**C**	Any immune-suppressing medication, such as Cyclosporine, FK 506, or Methotrexate [IMM]	1	2	7	9
	→**IF YES**, specify: [IMM_SPEC]				
**D**	Any immune-suppressing treatments, such as radiation therapy or chemotherapy [TX]	1	2	7	9
	→**IF YES**, specify: [TX_SPEC]				
**E**	Any stomach acid-reducing medications<br>→**IF YES**, did you take any of the following: [ACP]	1	2	7	9
	E1. Pepcid, Tagamet, Zantac, or Axid [ACP1]	1	2	7	9
	E2. Prilosec, Losec, Zoton, Protium or Nexium [ACP2]	1	2	7	9
**F**	Any other stomach acid-reducing medication [ACP_OTH]	1	2	7	9
	→**IF YES**, specify: [ACP_OTH_SPEC]				
**G**	Any other medications, not including antibiotics [OTH]	1	2	7	9
	→**IF YES**, specify: [OTH_SPEC]				

11. During the 3 weeks before **diarrhea [or other symptom]** began, *which was from __/__/_____ **(3 weeks before)** to __/__/_____ **(ONSET DATE)**,* did [you / your child] take antibiotics for an illness or any other reason? **(Note: If onset date was not recalled, use specimen collection date)** [PMU_ABX]

Yes.. 1
No.......................................**Go to Q.13**... 2

Don't know/Not sure........… **Go To Q.13**................................…...................... 7
Refused................................**Go To Q.13**... 9

"I am going to ask you some questions about the antibiotics [you were / your child was] taking during the ***three weeks before diarrhea [or other symptom] began****"*

12. A. What were the names of the antibiotics [you / your child] took **before diarrhea [or other symptom] began***?*
(REFER TO ANTIBIOTIC CODES IN ADMIN PACKET,
REPEAT QUESTIONS IF MULTIPLE ANTIBIOTICS ARE LISTED)
B. What date did [you / your child] start taking the antibiotic?
C. What was the last date that [you / your child] took the antibiotic?
D. About how many doses of antibiotic did [you / your child] miss?
E. Why [were you / was your child] taking this antibiotic? **(READ CHOICES, ENTER NUMBER IN BOX)**

Ear/sinus/upper respiratory infection… 1
Bronchitis or pneumonia……………….2
Urinary tract infection……………… …3
Skin infection………………………… 4
Diarrhea……………………………… 5
Other……………………. 6
Don't Know…………….. 7 **Do not read**
Refused…………………. 9 **Do not read**

F. How was this antibiotic obtained? **(READ CHOICES, ENTER NUMBER IN BOX)**

Prescribed for the problem that [you / your child] took it….1
Borrowed from friend or relative……..………2
Prescribed in the past for another problem….. 3
Other……………… 4
Don't Know……… 7 **Do not read**
Refused……………. 9 **Do not read**

**A. Antibiotic Name**	**CODE**	**B. Start date (999999 if DK mm/dd/yy**	**C. Last took (777777= still taking, 999999= DK) mm/dd/yy**	**D. How many missed doses?**	**E. Why took**	**F. How obtained**
Ab1: [ABX1]		[ABX1_ST]	[ABX1_STP]	[ABX1_MIS]	[ABX1_WHY]	[ABX1_OBT]
Ab2: [ABX2]		[ABX2_ST]	[ABX2_STP]	[ABX2_MIS]	[ABX2_WHY]	[ABX2_OBT]
Ab3: [ABX3]		[ABX3_ST]	[ABX3_STP]	[ABX3_MIS]	[ABX3_WHY]	[ABX3_OBT]
Other1: [OTH1]	52	[OTH1_ST]	[OTH1_STP]	[OTH1_MIS]	[OTH1_WHY]	[OTH1_OBT]
Other2: [OTH2]	53	[OTH2_ST]	[OTH2_STP]	[OTH2_MIS]	[OTH2_WHY]	[OTH2_OBT]
Don't Remember Name [UNK]	99	[UNK_ST]	[UNK_STP]	[UNK_MIS]	[UNK_WHY]	[UNK_OBT]

13A. Thinking specifically about the time period when [your / your child's] **diarrhea [or other symptom]** began, on ____/____/__________ (ONSET DATE), did [you / your child] **begin taking antibiotics for this illness before seeing a doctor or another health professional?** [P_ABX]

Yes.................................. 1 Don't know/Not sure...**Go To Q.13B**.... 7
No.........**Go to Q.13B**........2 Refused.........**Go To Q.13B**................. 9

A. What is the name of the antibiotic(s) [you / your child] took for **diarrhea [or other symptom]**, before seeing a doctor or another health professional? **(REFER TO ANTIBIOTIC CODES IN ADMIN PACKET, REPEAT FOR EACH ANTIBIOTIC)**
B. What date did [you / your child] start taking the antibiotic?
C. What was the last date that [you / your child] took the antibiotic?
D. How did you obtain this antibiotic? **((READ CHOICES, ENTER NUMBER IN BOX)**

Borrowed from friend or relative……………………..… 1
Prescribed in the past for another problem…………… 2
Other……………………………………………..…… 3
Don't Know……… 7 **Do not read**
Refused……………. 9 **Do not read**

A. Antibiotic Name	CODE	B. Start date? (999999 if unknown)	C. Last took? (777777=still taking, 999999= unknown)	D. How obtained
Ab1: [P_ABX1]		[P_ABX1_ST]	[P_ABX1_STP]	[P_ABX1_OBT]
Ab2: [P_ABX2]		[P_ABX2_ST]	[P_ABX2_STP]	[P_ABX2_OBT]
Ab3: [P_ABX3]		[P_ABX3_ST]	[P_ABX3_STP]	[P_ABX3_OBT]
Other1: [P_OTH1]	52	[P_OTH1_ST]	[P_OTH1_STP]	[P_OTH1_OBT]
Other2: [P_OTH2]	53	[P_OTH2_ST]	[P_OTH2_STP]	[P_OTH2_OBT]
Don't Remember Name [P_UNK]	99	[P_UNK_ST]	[P_UNK_STP]	[P_UNK_OBT]

13B. (IF NO ANTIBIOTICS WERE LISTED IN Q12 OR Q13A, GO TO Q14)

(IF ANTIBIOTICS WERE LISTED IN Q12 AND RESPONDENT COULD NOT REMEMBER THE NAME OR START DATE OR Q13 AND RESPONDENT COULD NOT REMEMBER THE NAME, COMPLETE INFORMATION IN BOX TO FACILITATE COMPLETION OF "ANTIBIOTIC FOLLOW-UP" FORMS FOR EACH ANTIBIOTIC) {There will be a maximum of 9 antibiotics from Question 12 and 13. The variable names for the antibiotics 3-9 will be the same as listed below except that the antibiotic number will be detailed by the number 1-9 after the variable name 'Ques'.}

"Can you tell me the name of the medical provider, ER, or clinic where you were prescribed the antibiotics, including the address and phone number if known, and the date the antibiotic was prescribed for each of the antibiotics that [you / your child] took?"

(APPROXIMATE DATE PRESCRIBED IF NECESSARY, IF RESPONDENT DOES NOT KNOW ADDRESS AND PHONE NUMBER, LOOK UP AFTER THE INTERVIEW RATHER THAN ASKING RESPONDENT TO FIND INFORMATION)

Question: 12 OR 13 [QUES1_Y]
Antibiotic: [QUES1_CO]
Date prescribed: [QUES1_PER]
Physician name: ~~[QUES1_PHY]~~
Clinic/ER name: ~~[QUES1_CLN]~~
Address: ~~[QUES1_ADD]~~
City, State [QUES1_CTY], [QUES1_ST]
Phone: ~~[QUES1_PHO]~~

Question: 12 OR 13 [QUES2_Y]
Antibiotic: [QUES2_CO]
Date prescribed: [QUES2_PER]
Physician name: ~~[QUES2_PHY]~~
Clinic/ER name: ~~[QUES2_CLN]~~
Address: ~~[QUES2_ADD]~~
City, State [QUES2_CTY], [QUES2_ST]
Phone: ~~[QUES2_PHO]~~

SECTION B-3: "*The next few questions are about visits that [you / your child] made to medical providers, for example, a doctor's office, clinic, urgent care clinics, or emergency rooms, for this illness. I am not referring to being admitted to a hospital overnight."*

14. How many times [have you / has your child] visited a doctor or other health professional **for this illness**? Please include clinic visits, urgent care clinic, emergency room and follow-up visits.

__ __ visits [MC_MP]
Don't know/Not sure.. 77
Refused.. 99

15. Can you tell me the name of the physician, name of the ER or clinic, address and phone number if known, and the date of the first visit for each medical provider [you / your child] visited for this illness?
(IF MULTIPLE VISITS TO SAME MEDICAL PROVIDER, LIST NUMBER OF VISITS, IF RESPONDENT DOES NOT KNOW ADDRESS AND PHONE NUMBER, LOOK UP AFTER THE INTERVIEW RATHER THAN ASKING RESPONDENT TO FIND INFORMATION)

Medical provider/facility #1 # Visits: ______[MC_MP1]______
Date of first visit: ____/____/______ (mm/dd/yy) [MP1_DT]
Physician name: _______~~[MP1_PHY]~~ __________
Clinic/ER name: _______~~[MP1_CLN]~~__________
Address: _______~~[MP1_ADD]~~__________ __
City, State: _______[MP1_CTY], [MP1_ST]__
Phone: _______~~[MP1_PHO]~~____________

Medical provider/facility #2 # Visits: __[MC_MP2]__
Date of first visit: ____/____/___ (mm/dd/yy) [MP2_DT]
Physician name: __________~~[MP2_PHY]~~_______
Clinic/ER name: __________~~[MP2_CLN]~~_______
Address: __________~~[MP2_ADD]~~_______
City, State: _____[MP2_CTY], [MP2_ST]____
Phone: ___________~~[MP2_PHO]~~______

Medical provider/facility #3 # Visits: ______[MC_MP3]______
Date of first visit: ____/____/______ (mm/dd/yy) [MP3_DT]

Medical provider/facility #4 # Visits: __[MC_MP4]__
Date of first visit: ____/____/___ (mm/dd/yy) [MP4_DT]

Physician name: ~~[MP3_PHY]~~
Clinic/ER name: ~~[MP3_CLN]~~
Address: ~~[MP3_ADD]~~
City, State: [MP3_CTY], [MP3_ST]
Phone: ~~[MP3_PHO]~~

Physician name: ~~[MP4_PHY]~~
Clinic/ER name: ~~[MP4_CLN]~~
Address: ~~[MP4_ADD]~~
City, State: [MP4_CTY], [MP4_ST]
Phone: ~~[MP4_PHO]~~

(COMPLETE "MEDICAL PROVIDER FOLLOW-UP" FORM FOR EACH MEDICAL PROVIDER)

16. [Were you / Was your child] admitted to a hospital overnight for this illness? [HSP]

Yes.. 1
No..........**Go to Q19**.................................. 2
Don't know/Not sure.......**Go to Q19**..................... 7
Refused.........**Go to Q19**............................... 9

→**IF YES,** How many times [were you / was your child] **admitted** to a hospital for this illness?
__ __ times [MC_HSP]
Don't know/Not sure.. 77
Refused.. 99

17. Can you tell me the name(s), address(es) and phone number(s), and the first date of admission for each of the hospitals where [you were / your child was] hospitalized for this illness?

(LIST EACH HOSPITAL WITH FIRST ADMISSION DATE: IF ADMIT DATE= Don't know/Not sure, ENTER 777777, IF ADMIT DATE= Refused, ENTER 999999; IF RESPONDENT DOES NOT KNOW THE ADDRESS AND PHONE NUMBER, LOOK UP AFTER THE INTERVIEW RATHER THAN ASKING RESPONDENT TO FIND INFORMATION)

Hospital #1 **First admit date:** _[HSP1_DT]___
Hospital Name/CODE: ~~[HSP1_NM]~~
Address: ~~[HSP1_ADD]~~
City, State: [HSP1_CTY],[HSP1_ST]
Phone: ~~[HSP1_PHO]~~

Hospital #2 **First admit date:** _[HSP2_DT]___
Hospital Name/CODE: ~~[HSP2_NM]~~
Address: ~~[HSP2_ADD]~~
City, State: [HSP2_CTY],[HSP2_ST]
Phone: ~~[HSP2_PHO]~~

Hospital #3 **First admit date:** _[HSP3_DT]___
Hospital Name/CODE: ~~[HSP3_NM]~~
Address: ~~[HSP3_ADD]~~
City, State: [HSP3_CTY],[HSP3_ST]
Phone: ~~[HSP3_PHO]~~

Hospital #4 **First admit date:** _[HSP4_DT]___
Hospital Name/CODE: ~~[HSP4_NM]~~
Address: ~~[HSP4_ADD]~~
City, State: [HSP4_CTY],[HSP4_ST]
Phone: ~~[HSP4_PHO]~~

(COMPLETE "HOSPITAL FOLLOW-UP" FORM FOR EACH HOSPITAL)

18. How many nights [were you / was your child] hospitalized for this illness? __ __ __ nights [HSP1_NIT], [HSP2_NIT], [HSP3_NIT], [HSP4_NIT]

Still hospitalized.................................. 777
Don't know/Not sure............................ 999

PART B-4: "*This is the last section of questions*."

19. At any time during this illness, did [you / your child] take …..?

**PLEASE READ EACH MEDICATION**			**Start date? (999999=DK) mm/dd/yy**	**Stop date? (777777=still taking, 999999= DK mm/dd/yy**
A	Any medicine for fever or pain	Y(1) N(2) DK(7) REF(9) [RX_PAIN]	[RX_PAIN_ST]	[RX_PAIN_STP]
	→**IF YES**, specify [RX_PAIN_SPEC]			
B	Pepto-Bismol	Y(1) N(2) DK(7) REF(9) [RX_PEPTO]	[RX_PEPTO_ST]	[RX_PEPTO_STP]
C	Kaopectate	Y(1) N(2) DK(7) REF(9) [RX_KAO]	[RX_KAO_ST]	[RX_KAO_STP]

D	Imodium	Y(1) N(2) DK(7) REF(9) [RX_IMOD]	[RX_IMOD_ST]	[RX_IMOD_STP]
E	Lomotil	Y(1) N(2) DK(7) REF(9) [RX_LOMO]	[RX_LOMO_ST]	[RX_LOMO_STP]
F	Other anti-diarrhea medication?	Y(1) N(2) DK(7) REF(9) [RX_ADA]	[RX_ADA_ST]	[RX_ADA_STP]
	→**IF YES**, specify [RX_ADA_SPEC]			

20. Did [you / your child] take any antibiotics or get treated with antibiotics for this illness after seeking medical care? This could be antibiotics taken on your own, antibiotics given during an office visit, antibiotics that were prescribed by a doctor or health professional, or antibiotics taken during or after hospitalization. [I_ABX]

Yes... 1 No...........**Go to Q23**................. 2
Don't know/Not sure.......**Go to Q23**...... 7 Refused...**Go toQ23**…………..... 9

"I am going to ask you some questions about the antibiotics [you / your child] took as a result of this illness"

21. A. What are the names of the antibiotics [you / your child] took?
(REFER TO ANTIBIOTIC CODES IN ADMIN PACKET, REPEAT FOR EACH ANTIBIOTIC)
B. What date did [you / your child] start taking the antibiotic?
C. What was the last date that [you / your child] took the antibiotic?
D. About how many doses of antibiotic did [you / your child] miss?
E. Was the antibiotic? **(READ EACH CHOICE, ENTER NUMBER IN BOX)**

Given during an office visit……………………………………………. 1
Prescribed by a doctor or health professional unrelated to hospitalization.... 2
Taken while hospitalized for this illness………………………………… 3
Prescribed at discharge from hospital………………………..……….. 4
Other………………………………………………………………….. 5, If other, specify
Don't Know………………………………………… 7 **Do not read**
Refused……………………………………………… 9 **Do not read**

**A. Antibiotic Name**	**CODE**	**B. Start date (999999 if unk), mmddyy**	**C. Last took (777777= still taking, 999999= unk) mm/dd/yy**	**D. How many missed doses?**	**E. How obtained (List all that apply)**	**Specify other**
Ab1: [I_ABX1]		[I_ABX1_ST]	[I_ABX1_STP]	[I_ABX1_MIS]	[I_ABX1_OBT]	[I_ABX1_OTH]
Ab2: [I_ABX2]		[I_ABX2_ST]	[I_ABX2_STP]	[I_ABX2_MIS]	[I_ABX2_OBT]	[I_ABX2_ OTH]
Ab3: [I_ABX3]		[I_ABX3_ST]	[I_ABX3_STP]	[I_ABX3_MIS]	[I_ABX3_OBT]	[I_ABX3_ OTH]
Other1: [I_OTH1]	52	[I_OTH1_ST]	[I_OTH1_STP]	[I_OTH1_MIS]	[I_OTH1_OBT]	[I_OTH1_ OTH]
Other2: [I_OTH2]	53	[I_OTH2_ST]	[I_OTH2_STP]	[I_OTH2_MIS]	[I_OTH2_OBT]	[I_OTH2_ OTH]
Don't Remember Name [I_UNK]	99	[I_UNK_ST]	[I_UNK_STP]	[I_UNK_MIS]	[I_UNK_OBT]	[I_UNK_ OTH]

22. Were any antibiotics [you were / your child was] taking for this illness discontinued before the full course for any reason? [I_DIS]

Yes.................... 1 Don't know/Not sure.......... 7
No..................... 2 Refused.............................. 9

→**IF YES, Which antibiotics:** ___________[I_DIS_SPEC]____________________
→**IF YES,** Why were the antibiotics stopped? (**READ EACH CHOICE**) [I_DIS_WHY]

Because of the diagnosis of *E. coli* O157 infection…………………………… 1
Because of suspicion or diagnosis of kidney problems, also called HUS…..… 2
Other reason…………………........……………….…................................ 3
Specify:_______________ [I_DIS_OTH]_____________________
Don't know/Not sure..………………....7 **Do not read**
Refused................ ………..……......9 **Do not read**

23. During this illness [were you / was your child] diagnosed with a kidney or blood problem, called hemolytic uremic syndrome or HUS? [DX_HUS]

Yes.................... 1 Don't know/Not sure.......... 7

No..................... 2 Refused............................... 9

→ **IF YES**, on what date was HUS diagnosed? _____[DX_HUS_DT]_____

mo day yr

Don't know/Not sure..................... 777777

Refused... 999999

24. During this illness [were you / was your child] diagnosed with thrombotic thrombocytopenic purpura or TTP? [DX_TTP]

Yes.................... 1 Don't know/Not sure.......... 7

No..................... 2 Refused............................... 9

→ **IF YES**, on what date was TTP diagnosed? _____[DX_TTP_DT]____

mo day yr

Don't know/Not sure........................777777

Refused...999999

"That was the last question."

IF THIS INTERVIEW IS LESS THAN 3 WEEKS (21 DAYS) AFTER SPECIMEN COLLECTION DATE, SCHEDULE A FOLLOW UP INTERVIEW:

"Could I schedule a brief interview with you in approximately 2 weeks to ask some follow up questions?"

Yes.................... 1 No..................... 2 [INT_SCHED]

IF YES, SCHEDULE FOLLOW UP INTERVIEW AT LEAST 3 WEEKS (21 DAYS) AFTER SPECIMEN COLLECTION DATE ALLOWING A MINIMUM OF 2 WEEKS BETWEEN THIS INTERVIEW AND THE FOLLOW UP INTERVIEW

Date of follow up interview: ____/____/____ [INT_FU] Time: ____[INT_TIME]________

mo day yr

"Thank you for your help with this important study."

IF NO, *"Thank you for your help with this important study."*

<u>SECTION C:</u> Interviewer information

1. Date of interview ____/____/____ [INT_DT]

mo day yr

2. Name of interviewer ____[INT_NM]________________ Name of person interviewed____________[INT_ID]______

3. Did you interview the case-patient? [INT_CS]

Yes.. 1

No.. 2

IF NO, Who completed the interview? [INT_PROXY]

Spouse/Partner…………………………………………. 1

Parent………………………………………………….... 2 →**CIRCLE:** FATHER **OR** MOTHER [INT_PROXY_PAR]

Guardian…….…………………………………….……. 3

Other Relative………………………………………….. 4

Other…………………………………………………… 5

→**SPECIFY**__________[INT_PROXY_OTH]____________

Don't Know/Not Sure…………………………………. 9

4. Complete all follow-up forms

		Need to complete?	How many forms need to be completed?
**A**	**ANTIBIOTIC FOLLOW-UP**<br>**Complete for each antibiotic for which name or start date is unk; Q 12 and 13**	YES NO<br>[AB_FU]	_____ forms [AB_FORMS]<br>(one form for each antibiotic exposure)
**B**	**MEDICAL PROVIDER FOLLOW-UP**<br>**Complete for each provider listed in question 15**	YES NO<br>[MED_FU]	_____ forms [MED_FORMS]<br>(one form per medical provider)
**C**	**HOSPITAL FOLLOW-UP**<br>**Complete for each hospital listed in question 17**	YES NO<br>[HOSPI_FU]	_____ forms [HOSPI_FORMS]<br>(one form per hospital)

<u>SECTION D:</u> FOLLOW-UP INTERVIEW

COMPLETE FOR CASE-PATIENTS WHOSE INITIAL INTERVIEW WAS LESS THAN 3 WEEKS (21 DAYS) AFTER SPECIMEN COLLECTION DATE, CONDUCT AT LEAST 3 WEEKS (21 DAYS) AFTER SPECIMEN COLLECTION DATE, ALLOW A MINIMUM OF TWO WEEKS BETWEEN THE INITIAL AND FOLLOW UP INTERVIEWS

REVIEW QUESTION REGARDING DURATION OF DIARRHEA, Q 8A PART A
REVIEW PREVIOUS QUESTIONS ABOUT ANTIBIOTICS (QUESTIONS 11-13, 20-22)

ATTEMPT TO INTERVIEW THE ILL PERSON OR THE PERSON THAT WAS INTERVIEWED INITIALLY

"Thank you for your participation in the first part of this study. I would like to ask a few questions to follow up on [your / your child's] illness."

→ IF Q8A-A= still having diarrhea, begin with Q1
→ IF diarrhea had stopped at the time of the first interview, begin with Q2

1. [Are you / Is your child] still having diarrhea? [FU_DA]

Yes........... 1 Don't know/Not sure.................... 7
No............ 2 Refused.. 9
→**IF NO,** how many days did the diarrhea last? ______ [FU_DA_DYS]

2. You indicated previously that [you / your child] **DID NOT TAKE / TOOK (QUESTIONS 11-13, 20-22)** antibiotics for **diarrhea [or other symptom]** or during the three weeks before this illness, before seeing a doctor or another health professional, or that were given during an office visit, antibiotics that were prescribed by a doctor or health professional, or antibiotics taken during or after hospitalization .

→ **DID NOT TAKE**, Is that correct? [FU_ABX_TK]

Yes.................... 1 Don't know/Not sure.......... 7
No..................... 2 Refused.............................. 9

→ (IF YES, GO TO 3)
→ (IF NO, REPEAT QUESTIONS BELOW)

During the 3 weeks before **diarrhea [or other symptom]** began, did [you / your child] take any antibiotics? [FU_ABX]

Y N DK REFUSED **→ IF YES, ASK QUESTION 12 FROM QUESTIONAIRE**

Did [you / your child] begin taking antibiotics for this illness before seeing a doctor or another health professional? [FU_PRE_ABX]

Y N DK REFUSED **→ IF YES, ASK QUESTION 13 FROM QUESTIONAIRE**

At any time during this illness did [you / your child] take an antibiotic? This could be antibiotics that were given during an office visit, antibiotics that were prescribed by a doctor or health professional, or antibiotics taken during or

after hospitalization. [FU_ABX_MED]
Y N DK REFUSED → **IF YES, ASK Q'S 21-22 FROM QUESTIONAIRE**

→ **TOOK, REFER TO ANTIBIOTIC QUESTIONS 12, 13, and 21; IF LAST DATE TAKEN FOR ANTIBIOTIC WAS UNKNOWN OR CASE WAS STILL TAKING ASK THE FOLLOWING FOR EACH ANTIBIOTIC**

When we last spoke, we were unable to record the last day that [you / your child] took the antibiotics.
A. When did [you / your child] stop taking _______________ (ANTIBIOTIC NAME)?
B. About how many antibiotic doses did [you / your child] miss?

**Antibiotic name**	**Question (circle)**	**A. Stop Date? (777777 = still taking, 999999= unknown)**	**B. How many?**
[FU_ NM1]	**12 13 21** [NM1_QUES]	[FU_ NM1_STP]	[FU_ NM1_DOSE]
[FU_ NM2]	**12 13 21** [NM2_QUES]	[FU_ NM2_STP]	[FU_ NM2_DOSE]
[FU_ NM3]	**12 13 21** [NM3_QUES]	[FU_ NM3_STP]	[FU_ NM3_DOSE]
[FU_ NM4]	**12 13 21** [NM4_QUES]	[FU_ NM4_STP]	[FU_ NM4_DOSE]

3. You indicated previously that [you were / your child was] **DIAGNOSED / NOT DIAGNOSED (Q23)** with a kidney or blood problem, also called hemolytic uremic syndrome or HUS during this illness.

→ **IF DIAGNOSED WITH HUS,** Is this correct? [FU_DX_HUS]
Yes.................... 1 Don't know/Not sure.......... 7
No..................... 2 Refused............................... 9
→**IF NO,** Could you explain the change in diagnosis?:__________[FU_HUS_CHANGE]____________________________

→ **IF NOT DIAGNOSED WITH HUS,** Is this still correct? [FU_DX_NOHUS]
Yes.................... 1 Don't know/Not sure.......... 7
No..................... 2 Refused............................... 9
→ **IF NO**, on what date was HUS diagnosed? ___[FU_DX_NOHUS_DT]___
mo day yr
Don't know/Not sure...……............777777
Refused…………….......................999999

4. You indicated previously that [you were / your child was] **DIAGNOSED / NOT DIAGNOSED (Q24)** with thrombotic thrombocytopenic purpura or TTP during this illness.

→ **IF DIAGNOSED WITH TTP,** Is this correct? [FU_DX_TTP]
Yes.................... 1 Don't know/Not sure.......... 7
No..................... 2 Refused............................... 9
→**IF NO,** Could you explain the change in diagnosis?:__________________ [FU_TTP_CHANGE]_________

→ **IF NOT DIAGNOSED WITH TTP,** Is this still correct? [FU_DX_NOTTP]
Yes.................... 1 Don't know/Not sure.......... 7
No..................... 2 Refused............................... 9
→ **IF NO**, on what date was TTP diagnosed? ____[FU_DX_NOTTP_DT]___
mo day yr
Don't know/Not sure...……............777777
Refused…………….......................999999

"That is all the questions that I have for you. We are very grateful for your time and effort in answering these questions. Again, thank you for participating."

SECTION E: Interviewer information

1. Date of follow-up interview ____/____/____, [FU_DT]
   mo day yr

2. Name of interviewer _________[FU_INTER]___________ Name of person interviewed_____[FU_PERSON]_______

3. Did you interview the same person that was interviewed initially (SECTION C, Question 2)? [FU_SAME]
   Yes.. 1
   No.. 2

   IF NO, Who completed the interview? [FU_WHO]
   Spouse/Partner………………………………………… 1
   Parent………………………………………………... 2 →**CIRCLE:** FATHER **OR** MOTHER[FU_PARENT]
   Guardian…….……………………………………..…… 3
   Other Relative……………………………………….. 4
   Other………………………………………………… 5 →**SPECIFY**___[FU_SPEC]__________________
   Don't Know/Not Sure…………………………………. 9

Section D: Follow up interview –Variable Key

12. A. What were the names of the antibiotics [you / your child] took **before diarrhea [or other symptom] began**?
(REFER TO ANTIBIOTIC CODES IN ADMIN PACKET,
REPEAT QUESTIONS IF MULTIPLE ANTIBIOTICS ARE LISTED)
B. What date did [you / your child] start taking the antibiotic?
C. What was the last date that [you / your child] took the antibiotic?
D. About how many doses of antibiotic did [you / your child] miss?
E. Why [were you / was your child] taking this antibiotic? **(READ CHOICES, ENTER NUMBER IN BOX)**

Ear/sinus/upper respiratory infection… 1
Bronchitis or pneumonia……………….2
Urinary tract infection……………… …3
Skin infection………………………… 4
Diarrhea……………………………… 5
Other……………………. 6
Don't Know…………….. 7 **Do not read**
Refused…………………. 9 **Do not read**

F. How was this antibiotic obtained? **(READ CHOICES, ENTER NUMBER IN BOX)**

Prescribed for the problem that [you / your child] took it….1
Borrowed from friend or relative……..………2
Other……………… 4
Don't Know……… 7 **Do not read**

**A. Antibiotic Name**	**CODE**	**B. Start date (999999 if DK mm/dd/yy**	**C. Last took (777777= still taking, 999999= DK) mm/dd/yy**	**D. How many missed doses?**	**E. Why took**	**F. How obtained**
Ab1: [FU_ABX1]		[FU_ABX1_ST]	[FU_ABX1_STP]	[FU_ABX1_MIS]	[FU_ABX1_WHY]	[FU_ABX1_OBT]
Ab2: [FU_ABX2]		[FU_ABX2_ST]	[FU_ABX2_STP]	[FU_ABX2_MIS]	[FU_ABX2_WHY]	[FU_ABX2_OBT]
Ab3: [FU_ABX3]		[FU_ABX3_ST]	[FU_ABX3_STP]	[FU_ABX3_MIS]	[FU_ABX3_WHY]	[FU_ABX3_OBT]
Other1: [FU_OTH1]	52	[FU_OTH1_ST]	[FU_OTH1_STP]	[FU_OTH1_MIS]	[FU_OTH1_WHY]	[FU_OTH1_OBT]
Other2: [FU_OTH2]	53	[FU_OTH2_ST]	[FU_OTH2_STP]	[FU_OTH2_MIS]	[FU_OTH2_WHY]	[FU_OTH2_OBT]
Don't Remember Name [FU_UNK]	99	[FU_UNK_ST]	[FU_UNK_STP]	[FU_UNK_MIS]	[FU_UNK_WHY]	[FU_UNK_OBT]

Prescribed in the past for another problem….. 3
Refused……………. 9 **Do not read**

13A. Thinking specifically about the time period when [your / your child's] **diarrhea [or other symptom]** began, on ____/____/__________ (ONSET DATE), did [you / your child] **begin taking antibiotics for this illness before seeing a doctor or another health professional?** [P_ABX]

Yes................................... 1
No.........**Go to Q.13B**........2
Don't know/Not sure...**Go To Q.13B**.... 7
Refused.........**Go To Q.13B**................. 9

A. What is the name of the antibiotic(s) [you / your child] took for **diarrhea [or other symptom]**, before seeing a doctor or another health professional? **(REFER TO ANTIBIOTIC CODES IN ADMIN PACKET, REPEAT FOR EACH ANTIBIOTIC)**
B. What date did [you / your child] start taking the antibiotic?
C. What was the last date that [you / your child] took the antibiotic?
D. How did you obtain this antibiotic? **((READ CHOICES, ENTER NUMBER IN BOX)**

Borrowed from friend or relative…………………..… 1
Prescribed in the past for another problem…………… 2
Other…………………………………………..…… 3
Don't Know……… 7 **Do not read**
Refused……………. 9 **Do not read**

A. Antibiotic Name<br><br><br>CODE		B. Start date? (999999 if unknown)	C. Last took? (777777=still taking, 999999= unknown)	D. How obtained
Ab1: [FU_P_ABX1]		[FU_P_ABX1_ST]	[FU_P_ABX1_STP]	[FU_P_ABX1_OBT]
Ab2: [FU_P_ABX2]		[FU_P_ABX2_ST]	[FU_P_ABX2_STP]	[FU_P_ABX2_OBT]
Ab3: [FU_P_ABX3]		[FU_P_ABX3_ST]	[FU_P_ABX3_STP]	[FU_P_ABX3_OBT]
Other1: [FU_P_OTH1]	52	[FU_P_OTH1_ST]	[FU_P_OTH1_STP]	[FU_P_OTH1_OBT]
Other2: [FU_P_OTH2]	53	[FU_P_OTH2_ST]	[FU_P_OTH2_STP]	[FU_P_OTH2_OBT]
Don't Remember Name [FU_P_UNK]	99	[FU_P_UNK_ST]	[FU_P_UNK_STP]	[FU_P_UNK_OBT]

21. A. What are the names of the antibiotics [you / your child] took*?*
(REFER TO ANTIBIOTIC CODES IN ADMIN PACKET, REPEAT FOR EACH ANTIBIOTIC)
B. What date did [you / your child] start taking the antibiotic?
C. What was the last date that [you / your child] took the antibiotic?
D. About how many doses of antibiotic did [you / your child] miss?
E. Was the antibiotic? **(READ EACH CHOICE, ENTER NUMBER IN BOX)**

Given during an office visit………………………………………………. 1
Prescribed by a doctor or health professional unrelated to hospitalization.... 2
Taken while hospitalized for this illness………………………………… 3
Prescribed at discharge from hospital………………………..……….. 4
Other……………………………………………………………………….. 5, If other, specify
Don't Know………………………………………… 7 **Do not read**
Refused……………………………………………… 9 **Do not read**

A. Antibiotic Name<br><br>CODE		B. Start date (999999 if unk), mmddyy	C. Last took (777777= still taking, 999999= unk) mm/dd/yy	D. How many missed doses?	E. How obtained (List all that apply)	Specify other
Ab1: [FU_I_ABX1]		[FU_I_ABX1_ST]	[FU_I_ABX1_STP]	[FU_I_ABX1_MIS]	[FU_I_ABX1_OBT]	[FU_I_ABX1_OTH]
Ab2: [FU_I_ABX2]		[FU_I_ABX2_ST]	[FU_I_ABX2_STP]	[FU_I_ABX2_MIS]	[FU_I_ABX2_OBT]	[FU_I_ABX2_OTH]
Ab3: [FU_I_ABX3]		[FU_I_ABX3_ST]	[FU_I_ABX3_STP]	[FU_I_ABX3_MIS]	[FU_I_ABX3_OBT]	[FU_I_ABX3_OTH]
Other1: [FU_I_OTH1]	52	[FU_I_OTH1_ST]	[FU_I_OTH1_STP]	[FU_I_OTH1_MIS]	[FU_I_OTH1_OBT]	[FU_I_OTH1_OTH]
Other2: [FU_I_OTH2]	53	[FU_I_OTH2_ST]	[FU_I_OTH2_STP]	[FU_I_OTH2_MIS]	[FU_I_OTH2_OBT]	[FU_I_OTH2_OTH]
Don't Remember Name [FU_I_UNK]	99	[FU_I_UNK_ST]	[FU_I_UNK_STP]	[FU_I_UNK_MIS]	[FU_I_UNK_OBT]	[FU_I_UNK_OTH]

22. Were any antibiotics [you were / your child was] taking for this illness discontinued before the full course for any reason? [FU_I_DIS]

Yes.................... 1   Don't know/Not sure.......... 7
No..................... 2   Refused.............................. 9

→**IF YES, Which antibiotics:** ___________[FU_I_DIS_SPEC]____________________
→**IF YES,** Why were the antibiotics stopped? (**READ EACH CHOICE**) [FU_I_DIS_WHY]

Because of the diagnosis of *E. coli* O157 infection……………………………. 1
Because of suspicion or diagnosis of kidney problems, also called HUS….…. 2
Other reason……………………........…………………..….................................. 3
Specify:_______________ [FU_I_DIS_OTH]______________________
Don't know/Not sure..………………....7 **Do not read**
Refused................ ……….……......9 **Do not read**
