# Supplementary material for: Risk of Hemolytic Uremic Syndrome Related to Treatment of Escherichia coli O157 Infection with Different Antimicrobial Classes
Source: Microorganisms. 2021 Sep 21;9(9):1997. doi: 10.3390/microorganisms9091997 (PMC8466573; doi:10.3390/microorganisms9091997)
Supplement: Supplementary file 1 [file microorganisms-09-01997-s001.zip › Medical Provider Form.pdf]

E. coli O157 COHORT STUDY: MEDICAL PROVIDER FOLLOW-UP

COMPLETE "MEDICAL PROVIDER FOLLOW-UP" FORM FOR EACH MEDICAL PROVIDER LISTED
CONTACT AT LEAST 3 WEEKS AFTER ILLNESS ONSET DATE

COHORT Study ID [COHORT_ID] __ __ __ __ __ **(2 letter state code and number, eg XX001)**

State Laboratory Isolate ID Number* ________[SLABS_ID]______ *This is required

Illness onset date _____[ILL_ONSET]_______

[**For Medical Provider Info variables, change "MP1" to "MP2", "MP3" or "MP4" for providers 2, 3, or 4, respectively.**]
Medical provider **1 2 3 4,** # Visits: ____[MC_MP1]____
Date of first visit: ___ [MP1_DT]____ (mm/dd/yy)
Physician name: _____ ~~[MP1_PHY]~~___________
Clinic/ER name: _____ [~~MP1_CLN~~]____________ **Address:** ________ ~~[MP1_ADD]~~________
City, State: ____ [MP1_CT], [MP1_ST]____ **Phone:** ________ ~~[MP1_PHO]~~________

[**For ALL variables below, add "_2", "_3", or "_4" to END of variable name for providers 2, 3, or 4, respectively.**]
(COMPLETE AFTER CONTACTING MEDICAL PROVIDER) Were you able to complete this "Medical provider follow-up" form for this medical provider? [MED_COM]
Yes....................................... 1, **IF YES**, Date completed __[MED_COM_DT]__ (mm/dd/yy)
No..2
→→**IF NO,** why were you not able to complete a "Medical provider Follow-Up" form? [MED_COM_RE]
Provider information incorrect.......................... 1
Provider refused access to records.................... 2
Records at provider unavailable........................ 3 →**SPECIFY**____[MED_COM_SPEC3]____________
Other.. 4 →**SPECIFY**____[MED_COM_SPEC4]____________

---

1. Date of the **first visit for this illness** to this medical provider? __[MED_FST_DT]__
mo day yr
Don't know/Not sure............777777

2. How many visits were made to this medical provider in the first 10 days of this illness? [MED_VS] __ __
Don't know/Not sure..................................... 77

3A. Patient blood type [MED_BLTYPE]
A………………………… 1
B………………………… 2
AB……………………… 3
O………………………………….. 4
Don't know/Not sure....................... 7

3B. Height: _[MED_HT]_ ft/in OR cm (circle), Date (closest to illness onset): __[MED_HT_DT]__ DK/Not sure.....777777

Weight(admit): _[MED_WT]_lbs OR kg (circle), Date (closest to illness onset): __[MED_WT_DT]_ DK/Not sure.....777777

4. A. What date was stool submitted for testing / culture by this medical provider? __[MED_STOOL]__
mo day yr
Don't know/Not sure..... 777777
Not done……**GO TO 5**… 999999

B. Was Shiga toxin testing performed? [MED_SHIGA] Yes.........1 No..........2 Don't know/Not sure.......... 7

→**IF YES,** →What was the result? [MED_SHIG_RES] Positive…1 Negative…2 Don't know/Not sure....... 7
→What date were results reported? __[MED_SHIG_DT]___
mo day yr
Don't know/Not sure.......... 777777

C. What date was *E. coli* O157 isolation reported from the lab? __[MED_ECOLI]__
mo day yr
Don't know/Not sure.......... 777777

5. Was a diagnosis of hemolytic uremic syndrome or HUS documented in the 3 weeks after the illness onset date? [MED_HUS]

Yes.................... 1 Don't know/Not sure.......... 7
No..................... 2
→ **IF YES**, on what date was HUS diagnosed? ___[MED_HUS_DT]___
mo day yr
Don't know/Not sure......... 777777

6. Was a diagnosis of thrombotic thrombocytopenic purpura or TTP documented in the 3 weeks after the illness onset date? [MED_TTP]

Yes.................... 1 Don't know/Not sure.......... 7
No..................... 2
→ **IF YES**, on what date was TTP diagnosed? __[MED_TTP_DT]__
mo day yr
Don't know/Not sure......... 777777

7. Were antibiotics given or prescribed for this illness? [MED_ABX]

Yes.................... 1 Don't know/Not sure.......... 7
No..................... 2
→ **IF YES, COMPLETE Q8**
→ **IF NO, Go to Q9**

8. **COMPLETE ROW FOR EACH ANTIBIOTIC DOCUMENTED IN CHART BY THIS PROVIDER**

A. AB names and codes **(ADMIN PACKET)**
B. Start date of the prescription or date given
C. Number of days of treatment prescribed or given? (eg 10 days, 14 days)
D. Was this antibiotic discontinued **because** of the diagnosis of *E. coli* O157?

A. Antibiotic Name	Ab Code	B. Start date (mmddyy)	C. # DAYS prescribed	D. Discontinued because of *E. coli* O157 diagnosis?
	[MED_ABX1]	[MED_ABX1_ST]	[MED_ABX1_DY]	Yes(1) No(2) DK(7) [MED_ABX1_DIS]
	[MED_ABX2]	[MED_ABX2_ST]	[MED_ABX2_DY]	Yes(1) No(2) DK(7) [MED_ABX2_DIS]
	[MED_ABX3]	[MED_ABX3_ST]	[MED_ABX3_DY]	Yes(1) No(2) DK(7) [MED_ABX3_DIS]
**OTH– SPECIFY** ______________	52 [MED_OTH1]	[MED_OTH1_ST]	[MED_OTH1_DY]	Yes(1) No(2) DK(7) [MED_OTH1_DIS]
**OTH– SPECIFY** ______________	53 [MED_OTH2]	[MED_OTH2_ST]	[MED_OTH2_DY]	Yes(1) No(2) DK(7) [MED_OTH2_DIS]

9. At the **first visit to this medical provider for this illness**, did the patient indicate a **history** of the following signs and/or symptoms?

A. Fever? [MED_FV] Yes(1) No(2) DK(7)
B. Nausea? [MED_NS] Yes(1) No(2) DK(7)
C. Vomiting? [MED_VM] Yes(1) No(2) DK(7)
D. Diarrhea? [MED_DA] Yes(1) No(2) DK(7)
E. Bloody stool? [MED_BLDST] Yes(1) No(2) DK(7)

10. Use the following charts to record symptoms, laboratory values, and treatment variables that are documented in medical records from this medical provider during the first **10 days of the illness**. This is from _____________ (Illness onset date) to ___________________ (9 days after illness onset date).

10. FOR EACH VISIT TO THIS MEDICAL PROVIDER THAT OCCURRED DURING THE FIRST 10 DAYS OF THIS ILLNESS (VISIT#1-4), RECORD THE DATE OF THE VISIT, SYMPTOMS, LABORATORY VALUES, AND INFORMATION ON TREATMENTS ON THAT DAY

A. Temperature? (Enter 777.7=DK, circle C or F)
B. Vomiting reported or documented on this day?
C. Diarrhea reported or documented on this day?
D. Blood (visible or occult) in stool reported or documented on this day?

E. BUN

F. Creatinine

G. LDH

H. Amylase

I. Lipase

J. Bilirubin

K. ALT

L. AST

M. Highest WBC count (per mm^3)

N. Hematocrit (%)

O. Hemoglobin

P. Platelet count (per mm^3)

Q. Blood smear performed, **IF yes,** Q1, Were erythrocyte fragments noted
IF yes, Specify (eg schistocytes, burr cells, helmet cells)

R. Urinalysis performed **IF yes**, J1, Hematuria?, **If yes**, Specify (eg 1+, 2+, 3+, 4+)
J2, Proteinuria? **If yes**, Specify (eg 1+, 2+, 3+, 4+)

S. Were IV fluids administered
If Yes, **S1.** Type of fluids
Normal saline……….1
½ Normal saline…….2
D5W………………..3
Other……………….4, **If other, specify other fluid type.**

S2. Volume (Liters / day)

T. Were blood products given
If Yes, Which products: T1. Packed cells T2. Whole blood T3. Platelets T4. Plasma

U. Anti-diarrhea medications given or prescribed (lomotil (diphenoxylate), loperamide, bismuth subsalicylate, kaolin pectin
If Yes, U1. Biologic agents: probiotics
U2. Anti-motility agents: lomotil (diphenoxylate), loperamide
U3. Adsorbent agents: bismuth subsalicylate, kaolin pectin

V. Other anti-diarrheal agents
If Yes, V1. Specify

Date	Visit #1<br>[MED_1]__/__/___	Visit #2<br>[MED_2]__/__/___	Visit #3<br>[MED_3]__/__/___	Visit #4<br>[MED_4]__/__/___
**SYMPTOMS**				
**A. Temp**	[MED_1_TEMP]<br>_ _ _ . _ F or C	[MED_2_TEMP]<br>_ _ _ . _ F or C	[MED_3_TEMP]<br>_ _ _ . _ F or C	[MED_4_TEMP]<br>_ _ _ . _ F or C
**B. Vomit**	[MED_1_VOMIT]<br>Y(1) N(2) DK(7)	[MED_2_VOMIT]<br>Y(1) N(2) DK(7)	[MED_3_VOMIT]<br>Y(1) N(2) DK(7)	[MED_4_VOMIT]<br>Y(1) N(2) DK(7)
**C. Diarrhea**	[MED_1_DA]<br>Y(1) N(2) DK(7)	[MED_2_DA]<br>Y(1) N(2) DK(7)	[MED_3_DA]<br>Y(1) N(2) DK(7)	[MED_4_DA]<br>Y(1) N(2) DK(7)
**D. Blood**	[MED_1_BLOOD]<br>Y(1) N(2) DK(7)	[MED_2_BLOOD]<br>Y(1) N(2) DK(7)	[MED_3_BLOOD]<br>Y(1) N(2) DK(7)	[MED_4_BLOOD]<br>Y(1) N(2) DK(7)

**LABORATORY VALUES**				
**E. BUN**	[MED_1_BUN]	[MED_2_BUN]	[MED_3_BUN]	[MED_4_BUN]
BUN unit	mg/dL or ___	mg/dL or ___	mg/dL or ___	mg/dL or ___
**F. Cr**	[MED_1_CR]	[MED_2_CR]	[MED_3_CR]	[MED_4_CR]
Cr unit	mg/dL or ___	mg/dL or ___	mg/dL or ___	mg/dL or ___
**G. LDH**	[MED_1_LDH]	[MED_2_LDH]	[MED_3_LDH]	[MED_4_LDH]
LDH unit	U/L or _____	U/L or _____	U/L or _____	U/L or _____
**H. Amylase**	[MED_1_AMY]	[MED_2_AMY]	[MED_3_AMY]	[MED_4_AMY]
unit (1) = U/L or (text)	[MED_1_AMY_U] or [MED_1_AMY_UO]	[MED_2_AMY_U] or [MED_2_AMY_UO]	[MED_3_AMY_U] or [MED_3_AMY_UO]	[MED_4_AMY_U] or [MED_4_AMY_UO]
**I. Lipase**	[MED_1_LIPASE]	[MED_2_LIPASE]	[MED_3_LIPASE]	[MED_4_LIPASE]
Lipase unit	U/L or ______	U/L or ______	U/L or ______	U/L or ______
**J. Bilirubin**	[MED_1_BIL]	[MED_2_BIL]	[MED_3_BIL]	[MED_4_BIL]
Bilirubin unit	mg/dL or ___	mg/dL or ___	mg/dL or ___	mg/dL or ___
**K. Transaminase Alanine (ALT)**	[MED_1_ALT]	[MED_2_ALT]	[MED_3_ALT]	[MED_4_ALT]
ALT unit	U/L or _____	U/L or _____	U/L or _____	U/L or _____
**L.** Transaminase Aspartate (AST)	[MED_1_AST]	[MED_2_AST]	[MED_3_AST]	[MED_4_AST]
AST unit	U/L or _____	U/L or _____	U/L or _____	U/L or _____
**M. WBC**	[MED_1_WBC]	[MED_2_WBC]	[MED_3_WBC]	[MED_4_WBC]
**N. Hct**	[MED_1_HCT]	[MED_2_HCT]	[MED_3_HCT]	[MED_4_HCT]
**O. Hb**	[MED_1_HEMO]	[MED_2_HEMO]	[MED_3_HEMO]	[MED_4_HEMO]
unit (1) = MG/DL or (text)	[MED_1_HEMOU] or [MED_1_HEMOOU]	[MED_2_HEMOU] or [MED_2_HEMOOU]	[MED_3_HEMOU] or [MED_3_HEMOOU]	[MED_4_HEMOU] or [MED_4_HEMOOU]
**P. Plt**	[MED_1_HPLAT]	[MED_2_HPLAT]	[MED_3_HPLAT]	[MED_4_HPLAT]
**Q. Blood smear**	[MED_1_BLOOD_SM]<br>Y(1) N(2) DK(7)	[MED_2_BLOOD_SM]<br>Y(1) N(2) DK(7)	[MED_3_BLOOD_SM]<br>Y(1) N(2) DK(7)	[MED_4_BLOOD_SM]<br>Y(1) N(2) DK(7)
**Q1 RBC frags**	[MED_1_RBC]<br>Y(1) N(2) DK(7)	[MED_2_RBC]<br>Y(1) N(2) DK(7)	[MED_3_RBC]<br>Y(1) N(2) DK(7)	[MED_4_RBC]<br>Y(1) N(2) DK(7)
**Q1 RBC specify**	[MED_1_RBC_SPEC]	[MED_2_RBC_SPEC]	[MED_3_RBC_SPEC]	[MED_4_RBC_SPEC]
**R. Urinalysis**	[MED_1_URINE]<br>Y(1) N(2) DK(7)	[MED_2_URINE]<br>Y(1) N(2) DK(7)	[MED_3_URINE]<br>Y(1) N(2) DK(7)	[MED_4_URINE]<br>Y(1) N(2) DK(7)
**R1 Hematuria**	[MED_1_HEMAT]<br>Y(1) N(2) DK(7)	[MED_2_HEMAT]<br>Y(1) N(2) DK(7)	[MED_3_HEMAT]<br>Y(1) N(2) DK(7)	[MED_4_HEMAT]<br>Y(1) N(2) DK(7)
**R1 Specify (circle or write)**	[MED_1_HEMAT_SPEC]<br>1+(1) 2+(2) 3+(3) 4+(4)	[MED_2_HEMAT_SPEC]<br>1+(1) 2+(2) 3+(3) 4+(4)	[MED_3_HEMAT_SPEC]<br>1+(1) 2+(2) 3+(3) 4+(4)	[MED_4_HEMAT_SPEC]<br>1+(1) 2+(2) 3+(3) 4+(4)
**R2 Proteinuria**	[MED_1_PROT]<br>Y(1) N(2) DK(7)	[MED_2_PROT]<br>Y(1) N(2) DK(7)	[MED_3_PROT]<br>Y(1) N(2) DK(7)	[MED_4_PROT]<br>Y(1) N(2) DK(7)
**R2 Specify (circle or write)**	[MED_1_PROT_SPEC]<br>1+(1) 2+(2) 3+(3) 4+(4)	[MED_2_PROT_SPEC]<br>1+(1) 2+(2) 3+(3) 4+(4)	[MED_3_PROT_SPEC]<br>1+(1) 2+(2) 3+(3) 4+(4)	[MED_4_PROT_SPEC]<br>1+(1) 2+(2) 3+(3) 4+(4)

INFORMATION ON TREATMENTS				
**S. IV fluids**	[MED_1_IV]<br>Y(1) N(2) DK(7)	[MED_2_IV]<br>Y(1) N(2) DK(7)	[MED_3_IV]<br>Y(1) N(2) DK(7)	[MED_4_IV]<br>Y(1) N(2) DK(7)
**S1. Type of fluids**	[MED_1_IV_TY]<br>1 2 3 4	[MED_2_IV_TY]<br>1 2 3 4	[MED_3_IV_TY]<br>1 2 3 4	[MED_4_IV_TY]<br>1 2 3 4
**Specify other fluids**	[MED_1_IV_OTH]	[MED_2_IV_OTH]	[MED_3_IV_OTH]	[MED_4_IV_OTH]
**S2. Volume (Liters/day)**	[MED_1_IV_VOL]	[MED_2_IV_VOL]	[MED_3_IV_VOL]	[MED_4_IV_VOL]
**T. Blood products**	[MED_1_BLD_PR]<br>Y(1) N(2) DK(7)	[MED_2_BLD_PR]<br>Y(1) N(2) DK(7)	[MED_3_BLD_PR]<br>Y(1) N(2) DK(7)	[MED_4_BLD_PR]<br>Y(1) N(2) DK(7)
**T1. Packed cells**	[MED_1_PCELLS]<br>Y(1) N(2) DK(7)	[MED_2_PCELLS]<br>Y(1) N(2) DK(7)	[MED_3_PCELLS]<br>Y(1) N(2) DK(7)	[MED_4_PCELLS]<br>Y(1) N(2) DK(7)
**T2. Whole blood**	[MED_1_WCELLS]<br>Y(1) N(2) DK(7)	[MED_2_WCELLS]<br>Y(1) N(2) DK(7)	[MED_3_WCELLS]<br>Y(1) N(2) DK(7)	[MED_4_WCELLS]<br>Y(1) N(2) DK(7)
**T3. Platelets**	[MED_1_PLAT]<br>Y(1) N(2) DK(7)	[MED_2_PLAT]<br>Y(1) N(2) DK(7)	[MED_3_PLAT]<br>Y(1) N(2) DK(7)	[MED_4_PLAT]<br>Y(1) N(2) DK(7)
**T4. Plasma**	[MED_1_PLASMA]<br>Y(1) N(2) DK(7)	[MED_2_PLASMA]<br>Y(1) N(2) DK(7)	[MED_3_PLASMA]<br>Y(1) N(2) DK(7)	[MED_4_PLASMA]<br>Y(1) N(2) DK(7)
**U. Anti-diarrheal**	[MED_1_ANTIDA]<br>Y(1) N(2) DK(7)	[MED_2_ANTIDA]<br>Y(1) N(2) DK(7)	[MED_3_ANTIDA]<br>Y(1) N(2) DK(7)	[MED_4_ANTIDA]<br>Y(1) N(2) DK(7)
**U1. Probiotic**	[MED_1_PROB]<br>Y(1) N(2) DK(7)	[MED_2_PROB]<br>Y(1) N(2) DK(7)	[MED_3_PROB]<br>Y(1) N(2) DK(7)	[MED_4_PROB]<br>Y(1) N(2) DK(7)
**U2. Anti-motility**	[MED_1_MOT]<br>Y(1) N(2) DK(7)	[MED_2_MOT]<br>Y(1) N(2) DK(7)	[MED_3_MOT]<br>Y(1) N(2) DK(7)	[MED_4_MOT]<br>Y(1) N(2) DK(7)
**U3. Adsorbent**	[MED_1_ADSORB]<br>Y(1) N(2) DK(7)	[MED_2_ADSORB]<br>Y(1) N(2) DK(7)	[MED_3_ADSORB]<br>Y(1) N(2) DK(7)	[MED_4_ADSORB]<br>Y(1) N(2) DK(7)
**V. Other anti-diarrheal**	MED_1_OTHER_ANTIDA<br>Y(1) N(2) DK(7)	MED_2_OTHER_ANTIDA<br>Y(1) N(2) DK(7)	MED_3_OTHER_ANTIDA<br>Y(1) N(2) DK(7)	MED_4_OTHER_ANTIDA<br>Y(1) N(2) DK(7)
**D1. Specify**	[MED_1_OTHER_SPEC]	[MED_2_OTHER_SPEC]	[MED_3_OTHER_SPEC]	[MED_4_OTHER_SPEC]
