# Supplementary material for: Risk of Hemolytic Uremic Syndrome Related to Treatment of Escherichia coli O157 Infection with Different Antimicrobial Classes
Source: Microorganisms. 2021 Sep 21;9(9):1997. doi: 10.3390/microorganisms9091997 (PMC8466573; doi:10.3390/microorganisms9091997)
Supplement: Supplementary file 1 [file microorganisms-09-01997-s001.zip › microorganisms-1367413-supplementary.pdf]

Supplementary Table S1. Demographic and clinical characteristics of study participants eligible for inclusion[a] in the primary multivariable adjusted models and participants excluded due to missing data.

Characteristic	Included (N=1155)	Excluded[b] (N=126)	*P* value[c]
Age, median (IQR), years	15 (5–37)	24 (7-52)	**0.01**[d]
Female (%)	596 (51.7)[e]	75 (59.5)	0.11
Confirmed HUS (%)	89 (7.7)	13 (10.3)	0.30
**Recorded antimicrobial exposure:**	**No. (%)**	**No. (%)**	
Any antibiotic	356 (30.8)	41 (32.5)	0.69
Fluoroquinolone	225 (19.5)	30 (23.8)	0.24
Metronidazole	191 (16.5)	19 (15.1)	0.80
Macrolide	12 (1.0)	0 (0)	0.62
β-lactam	55 (4.8)	3 (2.4)	0.36
Sulfonamide	29 (2.5)	5 (4.0)	0.37

Abbreviation: HUS, hemolytic uremic syndrome

[a] Some participants eligible for inclusion (i.e., had no missing covariate data) were still excluded from antimicrobial class-specific analyses if the participant was prescribed classes of antimicrobials other than the one of interest.

[b] The 126 participants excluded from the adjusted models were missing data for 1 or more covariates (acetaminophen (n=64), fever (n=60), vomiting (n=9)).

[c] Based on Fisher's exact test except where noted.

[d] Based on Wilcoxon rank-sum test.

[e] Sex was missing for one study participant.

Supplementary Table S2. Secondary models for the association between trimethoprim-sulfamethoxazole treatment during the first 7 days of illness and development of HUS.

Category[a]	HUS definition[b]	Source of antibiotic exposure data[c]	No. patients included	WBC included as model covariate?[d]	Adjusted OR[e]	95% CI
A	Confirmed or suspected	Documented or reported	789	No	1.80	0.75-4.34
				Yes	1.84	0.75-4.47
		Documented	846	No	2.43	0.96-6.14
				Yes	2.54	1.00-6.47
	Confirmed (suspected excluded)	Documented or reported	771	No	1.76	0.66-4.69
				Yes	1.76	0.66-4.69
		Documented	828	No [f]	2.37	0.85-6.60
				Yes	2.37	0.85-6.60
	Confirmed (suspected considered no HUS)	Documented or reported	789	No	1.58	0.60-4.17
				Yes	1.57	0.60-4.14
		Documented	846	No	2.05	0.75-5.61
				Yes	2.04	0.75-5.56
B	Confirmed (suspected excluded)	Documented or reported	464	No	1.99	0.69-5.75
				Yes	1.98	0.69-5.73
		Documented	483	No	2.53	0.83-7.72
				Yes	2.49	0.82-7.55
	Confirmed (suspected considered no HUS)	Documented or reported	481	No	1.67	0.59-4.74
				Yes	1.65	0.58-4.66
		Documented	500	No	2.02	0.69-5.96
				Yes	1.97	0.67-5.77

Abbreviations: HUS, hemolytic uremic syndrome; WBC, white blood cell count; CI, confidence interval.

[a] Models in category A assumed that test results for patients with missing results of any test used to define HUS would have been normal (i.e., not indicative of HUS) had they been performed. Models in category B excluded any patient missing data for any test used to define HUS (creatinine, platelets, hemoglobin or hematocrit, or peripheral blood smear).
[b] Confirmed HUS was defined as including all of the following abnormalities during the first 10 days of illness: 1) hemoglobin or hematocrit below age- and gender-specific thresholds, 2) fragmented erythrocytes on peripheral blood smear, 3) platelets <150 x10^9/L, and 4) serum creatinine ≥88.4 µmol/L if <13 years old or ≥132.6 µmol/L if ≥13 years old; Suspected HUS was defined as illness diagnosed by a treating clinician as HUS or thrombotic thrombocytopenic purpura in a patient with fragmented erythrocytes on peripheral blood smear, but lacking complete laboratory documentation required for a confirmed case.
[c] Documented exposure was defined as identification of antibiotic administration or prescription in medical records; reported exposure was defined as patient- (or legal guardian) reported exposure.
[d] In models that included the initial WBC value during the first 10 days of illness (and before HUS diagnosis) as a covariate, a dichotomous variable was used (WBC ≥17.2 x10^9/L versus <17.2 x10^9/L); patients with no WBC count documented were assumed to have WBC <17.2 x10^9/L.
[e] All models adjusted for age quartile, time to healthcare presentation, and patient- (or parent/guardian) reported fever, vomiting, and acetaminophen use.
[f] This is the primary model and these modelling assumptions were used for Table 3.

Supplementary Table S3. Secondary models for the association between metronidazole treatment during the first 7 days of illness and development of HUS.

**Category[a]**	**HUS definition[b]**	**Source of antibiotic exposure data[c]**	**No. patients included**	**WBC included as model covariate?[d]**	**Adjusted OR[e]**	**95% CI**
A	Confirmed or suspected	Documented or reported	971	No	1.42	0.57-3.54
				Yes	1.55	0.62-3.89
		Documented	1009	No	1.75	0.70-4.35
				Yes	1.86	0.75-4.65
	Confirmed (suspected excluded)	Documented or reported	952	No	1.40	0.53-3.74
				Yes	1.46	0.54-3.90
		Documented	990	No [f]	1.72	0.65-4.59
				Yes	1.76	0.66-4.70
	Confirmed (suspected considered no HUS)	Documented or reported	971	No	1.43	0.54-3.81
				Yes	1.46	0.55-3.90
		Documented	1009	No	1.75	0.66-4.65
				Yes	1.76	0.66-4.70
B	Confirmed (suspected excluded)	Documented or reported	624	No	1.38	0.50-3.79
				Yes	1.38	0.50-3.81
		Documented	633	No	1.30	0.48-3.56
				Yes	1.30	0.48-3.56
	Confirmed (suspected considered no HUS)	Documented or reported	642	No	1.40	0.51-3.83
				Yes	1.38	0.50-3.81
		Documented	651	No	1.32	0.49-3.60
				Yes	1.30	0.48-2.38

Abbreviations: HUS, hemolytic uremic syndrome; WBC, white blood cell count; CI, confidence interval.
[a] Models in category A assumed that test results for patients with missing results of any test used to define HUS would have been normal (i.e., not indicative of HUS) had they been performed. Models in category B excluded any patient missing data for any test used to define HUS (creatinine, platelets, hemoglobin or hematocrit, or peripheral blood smear).
[b] Confirmed HUS was defined as including all of the following abnormalities during the first 10 days of illness: 1) hemoglobin or hematocrit below age- and gender-specific thresholds, 2) fragmented erythrocytes on peripheral blood smear, 3) platelets <150 x10^9/L, and 4) serum creatinine ≥88.4 µmol/L if <13 years old or ≥132.6 µmol/L if ≥13 years old; Suspected HUS was defined as illness diagnosed by a treating clinician as HUS or thrombotic thrombocytopenic

purpura in a patient with fragmented erythrocytes on peripheral blood smear, but lacking complete laboratory documentation required for a confirmed case.
[c] Documented exposure was defined as identification of antibiotic administration or prescription in medical records; reported exposure was defined as patient- (or legal guardian) reported exposure.
[d] In models that included the initial WBC value during the first 10 days of illness (and before HUS diagnosis) as a covariate, a dichotomous variable was used (WBC ≥17.2 x10^9/L versus <17.2 x10^9/L); patients with no WBC count documented were assumed to have WBC <17.2 x10^9/L.
[e] All models adjusted for age quartile, time to healthcare presentation, and patient- (or parent/guardian) reported fever, vomiting, and acetaminophen use.
[f] This is the primary model and these modelling assumptions were used for Table 3.

Supplementary Table S4. Secondary models for the association between fluoroquinolone treatment during the first 7 days of illness and development of HUS.

**Category[a]**	**HUS definition[b]**	**Source of antibiotic exposure data[c]**	**No. patients included**	**WBC included as model covariate?[d]**	**Adjusted OR[e]**	**95% CI**
A	Confirmed or suspected	Documented or reported	1020	No	0.48	0.10-2.39
				Yes	0.49	0.10-2.41
		Documented	1042	No	0.28	0.04-2.11
				Yes	0.29	0.04-2.12
	Confirmed (suspected excluded)	Documented or reported	1002	No	0.57	0.11-3.02
				Yes	0.57	0.11-3.03
		Documented	1024	No [f]	0.30	0.04-2.53
				Yes	0.31	0.04-2.55
	Confirmed (suspected considered no HUS)	Documented or reported	1020	No	0.58	0.11-3.03
				Yes	0.58	0.11-3.04
		Documented	1042	No	0.30	0.04-2.54
				Yes	0.31	0.04-2.55
B	Confirmed (suspected excluded)	Documented or reported	645	No	0.52	0.10-2.80
				Yes	0.51	0.09-2.79
		Documented	650	No	0.24	0.03-2.04
				Yes	0.24	0.03-2.03
	Confirmed (suspected considered no HUS)	Documented or reported	662	No	0.51	0.09-2.76
				Yes	0.50	0.09-2.72
		Documented	667	No	0.24	0.03-2.01
				Yes	0.23	0.03-1.98

Abbreviations: HUS, hemolytic uremic syndrome; WBC, white blood cell count; CI, confidence interval.
[a] Models in category A assumed that test results for patients with missing results of any test used to define HUS would have been normal (i.e., not indicative of HUS) had they been performed. Models in category B excluded any patient missing data for any test used to define HUS (creatinine, platelets, hemoglobin or hematocrit, or peripheral blood smear).
[b] Confirmed HUS was defined as including all of the following abnormalities during the first 10 days of illness: 1) hemoglobin or hematocrit below age- and gender-specific thresholds, 2) fragmented erythrocytes on peripheral blood smear, 3) platelets <150 x10^9/L, and 4) serum creatinine ≥88.4 µmol/L if <13 years old or ≥132.6 µmol/L if ≥13 years old; Suspected HUS was defined as illness diagnosed by a treating clinician as HUS or thrombotic thrombocytopenic purpura in a patient with fragmented erythrocytes on peripheral blood smear, but lacking complete laboratory documentation required for a confirmed case.
[c] Documented exposure was defined as identification of antibiotic administration or prescription in medical records; reported exposure was defined as patient- (or legal guardian) reported exposure.
[d] In models that included the initial WBC value during the first 10 days of illness (and before HUS diagnosis) as a covariate, a dichotomous variable was used (WBC ≥17.2 x10^9/L versus <17.2 x10^9/L); patients with no WBC count documented were assumed to have WBC <17.2 x10^9/L.

[e] All models adjusted for age quartile, time to healthcare presentation, and patient- (or parent/guardian) reported fever, vomiting, and acetaminophen use.
[f] This is the primary model and these modelling assumptions were used for Table 3.
